# Pan-Genome-Based Characterization of the SRS Transcription Factor Family in Foxtail Millet

**DOI:** 10.3390/plants14081257

**Published:** 2025-04-21

**Authors:** Ruimiao Li, Cuiyun Lei, Qiang Zhang, Xiaomeng Guo, Xiting Cui, Xingchun Wang, Xukai Li, Jianhua Gao

**Affiliations:** Shanxi Hou Ji Laboratory, College of Life Sciences, Shanxi Agricultural University, Taigu, Jinzhong 030801, China; jiekexiaobai99@gmail.com (R.L.); cylei5116@163.com (C.L.); zq18295859941@163.com (Q.Z.); gxm2092120@163.com (X.G.); xt496989@163.com (X.C.); wxingchun@163.com (X.W.)

**Keywords:** foxtail millet, pan-genome, SRS transcription factors

## Abstract

The Short Internodes-Related Sequence (SRS) family, a class of plant-specific transcription factors crucial for diverse biological processes, was systematically investigated in foxtail millet using pan-genome data from 110 core germplasm resources as well as two high-quality genomes (*xm* and Yu1). We identified SRS members and analyzed their intra-species distribution patterns, including copy number variation (CNV) and interchromosomal translocations. A novel standardized nomenclature (*Accession_SiSRSN[.n]_xDy or xTy*) was proposed to unify gene family nomenclature, enabling the direct visualization of member number variation across germplasms and the identification of core/variable members while highlighting chromosomal translocations. Focusing on the two high-quality genomes, both harboring six core SRS members, we performed whole-genome collinearity analysis with Arabidopsis, rice, maize, soybean, and green foxtail. Ka/Ks analysis of collinear gene pairs revealed purifying selection acting on *SiSRS* genes. Promoter analysis identified abundant stress-responsive *cis*-elements. Among core members, the *xm_SiSRS5* gene exhibited the highest expression during vegetative growth but showed significant downregulation under drought and salt stress, suggesting its role as a key negative regulator in abiotic stress responses. This study demonstrates the utility of pan-genomics in resolving gene family dynamics and establishes *SiSRS5* as a critical target for stress tolerance engineering in foxtail millet.

## 1. Introduction

The Short Internodes-Related Sequence (SRS) transcription factor family, also referred to as the SHI (Short Internodes) family, represents a plant-specific group of transcriptional regulators that play pivotal roles in diverse developmental and stress-responsive processes. To date, SRS members have been identified across multiple species, including *Arabidopsis thaliana* [1,2], maize (*Zea mays*) [3], rice (*Oryza sativa*) [4], and barley (*Hordeum vulgare*) [5]. In *A. thaliana*, this family comprises 10 members, including SHI, STY1 (STYLISH1), STY2, LRP1 (Lateral Root Primordium 1), and SRS3–SRS8, that exhibit functional redundancy [2]. Structurally, SRS proteins are characterized by two conserved domains as follows: an N-terminal RING-like zinc finger domain and a C-terminal IXGH domain. The zinc finger domain harbors a C-X_2_-C-X_7_-C-X_4_-C-X_2_-C_2_-X_6_-C cysteine-rich motif. The IXGH domain contains a conserved “IXGH” (X means any amino acid) tetrapeptide motif enriched with acidic residues, facilitating homomeric or heteromeric dimerization [2,6]. Intriguingly, evolutionary analyses reveal that the IXGH domain has been lost in certain SRS members such as SRS8 (AT5G33210) [2].

SRS proteins regulate diverse plant development by modulating hormonal crosstalk. The *shi* mutant, a transposon-insertion mutant that leads to SHI overexpression in *A. thaliana*, exhibits gibberellin acid (GA)-insensitive dwarfism, delayed flowering, and twisted leaves, which are attributed to defects in GA biosynthesis, suggesting that SHI may act as a negative regulator of GA signaling [1,7]. STY1 directly activates *YUCCA4* (*YUC4*), a key gene in auxin biosynthesis, to orchestrate apical meristem formation during embryogenesis. This process is further modulated through auxin transport/signaling components such as PIN-FORMED 1 (PIN1) and PINOID (PID), highlighting the role of SRS proteins in coordinating auxin homeostasis to regulate developmental polarity [8]. STY1 and STY2 synergistically regulate gynoecium development in a dose-dependent manner, while higher-order mutants (e.g., quintuple mutants *sty1-1 sty2-1 shi-3 lrp1 srs5-1*) display severe phenotypic abnormalities, including disrupted apical tissue formation in gynoecia and enhanced leaf serration [1,2,6]. The expression of LRP1, which is involved in *A. thaliana* early lateral root formation by induction of the YUC4 expression, was identified during the initial stages of lateral and adventitious root primordia development and at the primary root primordium [9,10]. ZmLRP1 (GRMZM2G077752) plays an essential role in the formation of lateral roots and crown roots in maize. Its expression is directly repressed by RUM1 (Rootless with Undetectable Meristem 1) and Aux/IAA (Auxin/Indole-3-Acetic Acid) proteins by binding to the *ZmLRP1* promoter [11]. Additionally, *ZmLRP1* is the only *SRS* member with high expression level in senescing leaves, and it may activate carbohydrate remobilization via abscisic acid (ABA) signaling, underscoring the complexity of SRS–hormone interactions [3]. SRS5 promotes photomorphogenesis by activating *ELONGATED HYPOCOTYL5* (*HY5*) and *B-BOX PROTEIN* (*BBX21* and *BBX22*) genes while inhibiting lateral root formation by suppressing *LATERAL ORGAN BOUNDARIES-DOMAIN* (*LBD16* and *LBD29*) expression [12,13]. In barley, VRS2 balances gradients of auxin, cytokinin, and GA to regulate the spike architecture and floret patterning [5].

SRS proteins are also involved in abiotic stress responses, exhibiting species-specific patterns. Rice OsSHI1 integrates auxin, brassinosteroid (BR), and ABA signaling to coordinate growth-stress trade-offs [14]. The overexpression of soybean (*Glycine max*) *GmSRS18* in *A. thaliana* negatively regulates drought and salt tolerance [15], whereas five *OsSRSs* in rice are upregulated under salt and drought stress [4]. Cotton (*Gossypium hirsutum*) *GhSRS21* negatively modulates salt tolerance by regulating ROS (Reactive Oxygen Species) homeostasis [16]. In *Solanum lycopersicum*, eight *SlSRS* genes were identified and seven members showed responsiveness at a transcription level to different abiotic treatments, including dehydration, oxidative stress, salinity, drought, injury, and osmotic stress [17].

Despite these advances, the functional characterization of the SRS family in foxtail millet (*Setaria italica*), a drought-tolerant C_4_ model crop, remains unexplored. While pan-genomic studies have elucidated core-variable gene distributions (core genes present in most of all individuals; variable genes include copy-number variations (CNVs) and gene presence/absence variations (gPAVs) within species [18,19]), systematic analyses of SRS members in foxtail millet are lacking. Deciphering the roles of *SRS* genes in this resilient species could unveil the molecular mechanisms underlying stress adaptation and provide novel targets for crop improvement.

## 2. Materials and Methods

### 2.1. Data Acquisition

Genomic and proteomic data for *A. thaliana* SRS family members were retrieved from the TAIR database (https://www.arabidopsis.org/, accessed on 14 July 2024), and conserved domain features were annotated using InterPro (http://pfam-legacy.xfam.org/, accessed on 14 July 2024). Hidden Markov Models (HMMs) corresponding to SRS domains were downloaded for subsequent analysis. Protein sequences, genome annotations, and biological information for green foxtail (*Setaria viridis*), maize, rice, soybean, and *A. thaliana* were obtained from Phytozome (https://phytozome-next.jgi.doe.gov/, accessed on 14 July 2024). The mutant with a short life cycle of the foxtail millet elite cultivar Jingu21 *xiaomi* (*xm*) dataset was sourced from the *Setaria italica* Multi-Omics Database (http://sky.sxau.edu.cn/MDSi.htm, accessed on 15 July 2024) [20], while the Telomere-to-Telomere (T2T) assembled genome of the Yugu1 (Yu1) cultivar was acquired from *Setaria*-DB (http://111.203.21.71:8000/index.html, accessed on 1 January 2024) [21]. Transcriptomic data from 110 foxtail millet pan-genome accessions [22], Yu1, and *xm* were processed using Perl hash structures to extract primary protein-coding transcripts, which were compiled into a pan-genome protein database.

### 2.2. Identification and Phylogenetic Analysis of SRS Family Members

A dual approach combining BLASTP- and HMM-based screening was employed to comprehensively identify SRS homologs. First, 10 *A. thaliana* SRS protein sequences were used as queries for BLASTP (blast+, v2.13.0, National Center for Biotechnology Information, Bethesda, MD, USA) searches against protein databases containing *A. thaliana*, soybean, maize, rice, green foxtail, and foxtail millet (Yu1 and *xm*), with an E-value cutoff of <1 × 10^−5^. Second, an HMM profile specific to the SRS family was constructed from *A. thaliana* SRS using InterPro (https://www.ebi.ac.uk/interpro/, accessed on 21 July 2024) and applied to screen the same databases via HMMER (v3.3.2, Howard Hughes Medical Institute, Chevy Chase, MD, USA) [23] (E-value < 1 × 10^−5^). Candidate sequences from both methods were aligned using MAFFT (v7.505, Research Institute for Microbial Diseases, Osaka University, Osaka, Japan, L-INS-*i* algorithm) [24], and the alignment was iteratively refined with HMMER to reconstruct a high-confidence HMM. This refined model was reapplied to the databases under identical thresholds, and the intersection of BLASTP and HMM results yielded the final candidate *SRS* gene set.

For phylogenetic reconstruction, candidate protein sequences were aligned with MAFFT, and a maximum-likelihood tree was generated using IQ-Tree (v2.0.3, Center for Integrative Bioinformatics Vienna, Max F. Perutz Laboratories, University of Vienna, Vienna, Austria) [25] under the best-fit substitution model (automatically selected by ModelFinder). Branch support was assessed with UFboot (UltraFast bootstrap approximation, 1000 replicates) [26] and SH-aLRT (approximate likelihood ratio test) [27]. The resulting topology was visualized as a cladogram using iTOL (v7, Biobyte Solutions GmbH, Heidelberg, Germany, https://itol.embl.de/, accessed on 15 July 2024).

For pan-genome analyses, the same pipeline was applied, substituting the protein database with the pan-genome and the *xm* and Yu1 dataset. Subspecies-specific gene distributions across phylogenetic clades were quantified using R (v4.3.1, R Foundation for Statistical Computing, Vienna, Austria), and candidate genes were mapped to their genomic loci on the T2T assembly using Python (v3.9.11, Python Software Foundation, Wilmington, DE, USA). Visualization of chromosomal positions was performed with the R package Rideogram (v0.2.2) [28]. We constructed a phylogenetic tree of pan-genome gene family members using FastTree (v2.1.11, Lawrence Berkeley National Laboratory, Berkeley, CA, USA) [29].

### 2.3. Analysis of Promoter Sequences of SRS Family Members at the Pan-Genome Level

The upstream 2000 bp sequences of all SRS members in the 112 accessions were extracted using bedtools (v2.31.1, Quinlan Laboratory, University of Utah, Salt Lake City, UT, USA) and submitted to PlantCARE (http://bioinformatics.psb.ugent.be/webtools/plantcare/html/, accessed on 4 April 2025) for the prediction of their *cis*-regulatory element (CRE) distributions [30]. Visualization was performed using TBtools (v2.003) [31]. Predicted elements were categorized by function (e.g., abiotic stress response, phytohormone signaling, and plant growth and development) and visualized as a heatmap using the ComplexHeatmap (v2.22.0, Bioconductor Project, Dana-Farber Cancer Institute, Boston, MA, USA) R package [32].

### 2.4. Motif and Conserved Domain Analysis

Conserved motifs in *SiSRS* proteins were predicted using MEME Suite (https://meme-suite.org/meme/tools/meme, last accessed on 1 April 2025) [33], with parameters set to identify 6-100 aa motifs (maximum 10 motifs per sequence, E-value < 1 × 10^−5^). Conserved domains were annotated via NCBI’s Batch-CD-Search (https://www.ncbi.nlm.nih.gov/Structure/bwrpsb/bwrpsb.cgi, accessed on 1 April 2025) [34], using the default thresholds. Results were visualized using TBtools (v2.003) [30] to generate integrated diagrams of the motif-domain architectures.

### 2.5. Collinearity and Selective Pressure Analysis

The intra- and inter-species collinearity of *SRS* genes was analyzed using MCScanX (Plant Genome Mapping Laboratory, Institute of Bioinformatics, Department of Plant Biology, University of Georgia, Athens, GA, USA) [35]. For cross-species comparisons, reciprocal BLASTP (E-value < 1 × 10^−5^, num Hits = 10) was performed between foxtail millet (*xm* and Yu1) and reference species to identify syntenic gene pairs. Intra-species collinearity within foxtail millet was assessed using the Yu1 genome as the reference. To evaluate evolutionary selection pressure, Ka/Ks ratios for collinear *SiSRS* gene pairs were calculated with KaKs_Calculator (v3.0, National Genomics Data Center, Beijing Institute of Genomics, Chinese Academy of Sciences, Beijing, China) [36] with the NG86 model, which accounts for non-synonymous (Ka) and synonymous (Ks) substitution rates.

### 2.6. RNA-seq Data Mining

The expression profiles of *SiSRS* genes across spatiotemporal tissues were extracted from transcriptomic datasets. Expression values were normalized to TPM (transcripts per million), and a tissue-specific expression heatmap was generated using the pheatmap (v1.0.12, R Foundation for Statistical Computing, Vienna, Austria) R package [37]. Pairwise Pearson correlation coefficients of gene expression patterns were calculated and visualized with corrplot (v0.95, R Foundation for Statistical Computing, Vienna, Austria).

### 2.7. RT-qPCR Validation

*Stress treatments*: Twenty-one-day-old *xm* seedlings were subjected to drought (20% PEG6000, *w*/*v*) or salt stress (250 mM NaCl). Leaf and root tissues were sampled at 0, 6, 12, 24, 48, and 72 h post-treatment. Untreated seedlings at corresponding time points served as controls.

*RNA extraction and cDNA synthesis*: Total RNA was isolated using the Plant RNA Kit (R6827, OMEGA, Guangzhou, China). Genomic DNA was removed with the PrimerScript™ gDNA Eraser, and cDNA was synthesized using the PrimerScript™ RT Reagent Kit (RR047A, TaKaRa, Beijing, China).

*qPCR Amplification*: Reactions were performed on a Bio-Rad CFX96 system (Bio-Rad Laboratories, Hercules, CA, USA) in 10 µL volumes containing the following: 5 µL TB green premix Ex Taq II (2×), 0.5 µL forward/reverse primers (10 µM each), 0.2 µL ROX reference dye II (50×), 1 µL cDNA template, and 2.8 µL ddH_2_O. The thermal cycling conditions were as follows: initial denaturation at 95 °C for 30 s, followed by 40 cycles of denaturation at 95 °C for 5 s and annealing/extension at 60 °C for 34 s. Three biological replicates with technical triplicates were included for each sample.

### 2.8. Gene Co-Expression and Protein–Protein Interaction Networks

*Co-expression network analysis*: A weighted gene co-expression network was constructed using the WGCNA (v1.73, R Foundation for Statistical Computing, Vienna, Austria) R package [38]. Transcriptomic data were preprocessed to retain genes with non-zero variance and a sample missing rate < 10%. Soft thresholding power was determined via the scale-free topology criteria (R^2^ > 0.80). For each *SiSRS* gene, the top 50 co-expressed partners (ranked by connection weight) were selected to build sub-networks, which were visualized using Cytoscape (v3.10.2, The Cytoscape Consortium, San Diego, CA, USA) [39].

*Protein–Protein Interaction (PPI) network*: *SiSRS* protein sequences were queried against the STRING database (https://string-db.org/, accessed on 26 August 2024) [40] using *A. thaliana* as the reference species. Interactions with a confidence score > 0.70 were retained, and the network topology was refined to highlight high-confidence nodes (e.g., hubs with ≥5 edges). The final PPI network was imported into Cytoscape for layout optimization and functional annotation.

## 3. Results

### 3.1. Pan-Genome Distribution of the SiSRS Gene Family

**Figure 1:**
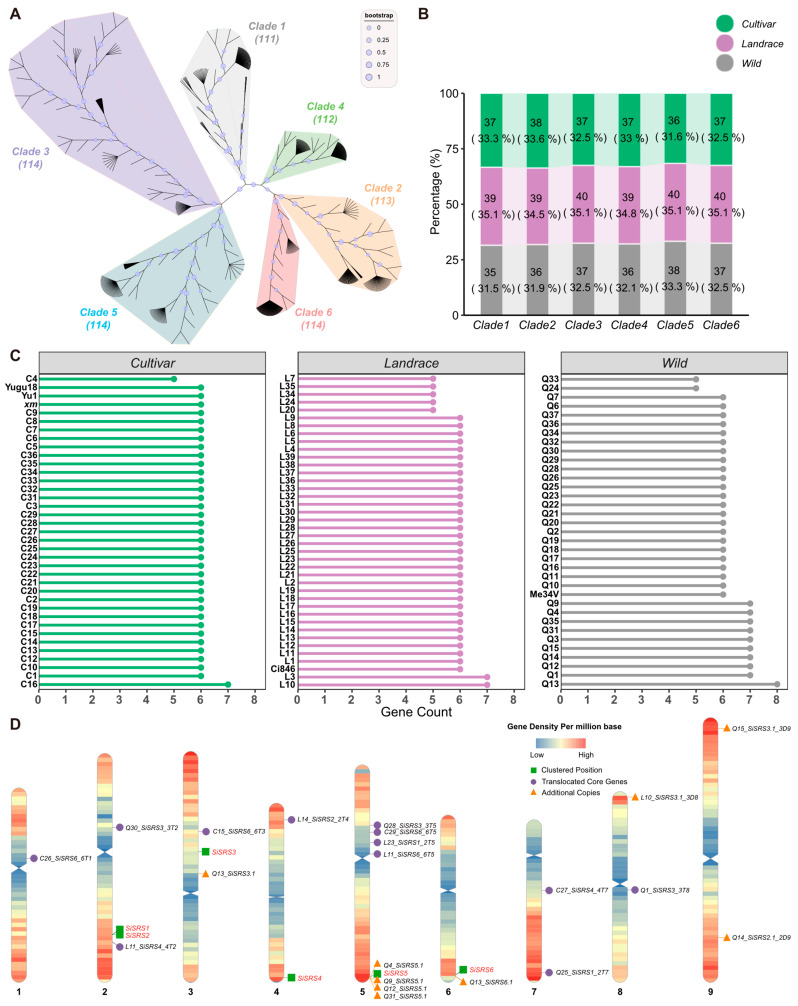
Identification and distribution analysis of *SiSRS* gene members in the 112 accessions. (**A**) Phylogenetic tree of pan-genome accession *SiSRSs*; (**B**) proportion of gene members from cultivated, landrace, or wild varieties in each of the six branches relative to the total number of genes in the corresponding branch; (**C**) *SRS* member count in each accession; (**D**) chromosomal location and the relative position of pan-genome *SRS* members. The bold numbers below each chromosome indicate the chromosome numbers.

Alongside the pan-genome dataset comprising 110 foxtail millet accessions (35 cultivars, 40 landraces, and 35 wild varieties), genomic resources from the elite cultivar Yu1 and the Jingu21-derived mutant line *xm* were integrated, resulting in a consolidated dataset of 112 accessions. Comprehensive analysis of the 112 accessions identified 678 *SiSRS* members, phylogenetically classified into six distinct clades (Clade 1-6) based on the encoded polypeptides (Figure 1A), suggesting there are six core genes in the species. The clade-specific member counts remained stable across subspecies (Figure 1B), with 91 accessions harboring all six core *SiSRS* genes (*SiSRS1-6*), confirming their conserved status (Figure 1C). These core genes exhibited conserved chromosomal localization as follows: *SiSRS1* and *SiSRS2* co-localized on chromosome 2 (Chr2), while *SiSRS3-6* mapped to chromosomes 3-6, respectively (Figure 1D).

Twelve accessions harbored a seventh *SiSRS* copy, and Q13 uniquely retained an eighth copy. These additional copies displayed high homology to specific core genes, suggesting the existence of recent duplication events (Figure 1C, Supplementary Table S1). C16, Q14, and Q35 shared near-identical sequences with *SiSRS2* (Chr2). However, Q14′s copy localized to Chr9, while C16/Q35 copies remained unanchored to chromosomes. L10 and Q15 duplicates showed highest homology to *SiSRS3* (Chr3) but were positioned on Chr8 and Chr9, respectively. L3 and Q1 duplicates aligned with *SiSRS4* (Chr4) but remained in unassembled regions. Q4, Q9, Q12, and Q31 duplicates clustered adjacently on Chr5, mirroring *SiSRS5*’s locus. Q3′s duplicate belonged to clade 6 but also remained unassembled. Q13 retained an extra *SiSRS6* copy on Chr6 and a *SiSRS3* duplicate on Chr3. Notably, no accessions contained additional *SiSRS1* copies, underscoring its evolutionary stability (Supplementary Table S2).

The core genes of *SiSRS* exhibit evidence of cross-chromosomal distribution. For instance, L23 contains six *SRS* genes, yet *SiSRS1* is located on Chr5 instead of the expected Chr2, while *SiSRS1* of Q25 has translocated from Chr2 to Chr7. Similarly, *SiSRS2* in accession L14 has translocated from Chr2 to Chr4. Additionally, *SiSRS3* in Q1, Q28, and Q30 is positioned on Chr8, 5, and 2, respectively, rather than Chr3. Gene *SiSRS4* in C27 and L11 has relocated to chromosomes 7 and 2, respectively. Furthermore, the *SiSRS6* gene in C15, C26, C29, and L11 is found on Chr3, 1, 5 and 5, respectively (Figure 1D, Supplementary Table S1). Moreover, certain accessions (C16, L3, Q1, Q3, and Q35) contain *SiSRS* genes that have not been assembled into specific chromosomal regions; however, these genes are still distributed across the six branches (Supplementary Table S2).

Additionally, eight accessions (C4, L7, L20, L24, L34, L35, Q24, and Q33) lack certain core members of the *SiSRS* family, containing only five *SRS* genes. Among these, L7 lacks *SiSRS1*, L34 lacks *SiSRS2*, L20 lacks *SiSRS3*, L24 and L35 lack *SiSRS4*, C4 and Q33 lack *SiSRS5*, and Q24 lacks *SiSRS6*. These deficiencies may be due to issues related to sequencing depth or annotation quality. The case of Me34V is particularly noteworthy as it lacks *SiSRS2* but possesses an additional copy of *SiSRS6* (Figure 1C, Supplementary Table S1).

### 3.2. Pan-Genome-Based Nomenclature for Gene Family Members

Based on previous analyses, this study has systematically established a naming convention for members of the *SiSRS* gene family at the pan-genome level. The proposed system is designed to provide clarity and consistency in gene nomenclature, facilitating the identification and classification of gene copies across different genomes. The naming format is outlined as follows:

*Core gene naming*: The naming of core members begins with the accession name as a prefix, followed by their chromosomal locations using the format “*SiSRSN*” (where *N* ranges from 1 to 6), separated by an underscore “_”. This numbering process remains consistent even if a specific member is absent in a particular germplasm. When describing the *SRS* core genes of the species, the accession name can be omitted, such as *SiSRS1*.

*Additional copies based on phylogenetic relationships*: Based on phylogenetic relationships, the additional copies of a core member are designated as ‘*1*′, ‘*2*′, etc., according to the chromosomal physical position order. That is, if there exists an *n*th additional copy, it is suffixed with ‘*.n*’. This approach ensures that each copy is uniquely identified based on its phylogenetic relationship to the core gene.

*Interchromosomal localization of gene copies*: The naming system emphasizes the interchromosomal localization of gene copies. After the name of a core gene, the chromosomal localization *x* followed an underscore, which can be omitted. For an additional copy, the primary chromosomal position *x* of the corresponding core gene with the highest homology is indicated after an underscore, followed by the actual chromosomal location *y* of the additional copy, separated by the letter “*D*”, i.e., “_*xDy*” (“*D*” means “duplicate of”). For genes that have not undergone interchromosomal insertional translocation and duplication relative to their corresponding core gene, the single value *x* suffices and may be omitted if there is no ambiguity. For core genes transferred to unexpected chromosomes, *x* represents the expected chromosome number, and *y* represents the actual chromosome number. These two values are connected by the letter “*T*”, i.e., “*_xTy*” (“*T*” means “transfer to”).

The complete naming convention is as follows:

*Accession_SiSRSN[.n]_xDy or xTy*

For example, in Q13, additional copies of *SiSRS3* and *SiSRS6* are denoted by *Q13_SiSRS3.1_3D3* (alternatively denoted as *Q13_SiSRS3.1_3* or *Q13_SiSRS3.1*), *Q13_SiSRS6.1_6D6* (alternatively denoted as *Q13_SiSRS6.1_6* or *Q13_SiSRS6.1*). In C26, the core gene *SiSRS6* has transferred from chromosome 6 to chromosome 1, which is named *C26_SiSRS6_6T1*. In L10, an additional copy of *SiSRS3* (Chr3) is located on chromosome 8, named *L10_SiSRS3.1_3D8*. In L34, although the core gene *SiSRS2* is missing, the other core genes retain their original numbering, i.e., *L34_SiSRS1_2*, *L34_SiSRS3_3*, *L34_SiSRS4_4*, *L34_SiSRS5_5*, and *L34_SiSRS6_6*. The core gene names of L34 can also be abbreviated, for instance, *L34_SiSRS1_2* can be written as *L34_SiSRS1*.

### 3.3. Pan-Genome-Based Promoter Analysis

The promoters of *SiSRSs* can also be classified into six phylogenetic branches, with each branch primarily representing the corresponding clade of *SRS* family, along with additional copies identified through evolutionary analysis based on amino acid sequences (Supplementary Figure S1). However, unexpected members from other branches were found within each phylogenetic group. For instance, the *SiSRS1* branch contains *Yu1_SiSRS2*, and the *SiSRS2* branch contains *Yu1_SiSRS1*, *Q18_SiSRS5*, *Q14_SiSRS5*, *Q13_SiSRS5*.

Regarding the distribution of CREs, differences exist between branches, and various distributions are also observed within each branch. For instance, the *SiSRS5* clade predominantly exhibits three distribution patterns (pattern5_1, pattern5_2, and pattern5_3). Intriguingly, pattern5_2 and pattern5_3 are exclusively detected in a subset of cultivated accessions and landraces, whereas all 35 wild accessions completely lacked these patterns in their *SiSRS5* promoters. Compared to pattern5_2, pattern5_3 demonstrates a 5′-ward shift, suggesting 3′-prime fragment insertion. Conversely, this phenomenon could alternatively result from regional fragment deletion in pattern5_2. Notably, pattern5_1 represents the predominant structural configuration observed in wild-type CREs, encompassing only sporadic cases of landrace varieties such as *L9_SiSRS5*, *L23_SiSRS5*, and *L25_SiSRS5*. The absence of explicit pattern specifications for certain accessions stems from these forms being only slight variations of existing patterns. For instance, *xm_SiSRS5* and *Yu1_SiSRS5* exhibit merely marginal deviations from the established pattern5_3 framework. Moreover, certain wild-type accessions (e.g., *Q31_SiSRS5* and *Q30_SiSRS5*) exhibit divergent spatial arrangements that deviate markedly from pattern5_1, from which no clear migration patterns could be discerned. The *SiSRS2* clade also demonstrates three patterns (pattern2_1, pattern2_2, and pattern2_3). Pattern2_1 and pattern2_2 comprehensively encompass the majority of wild accessions, whereas pattern2_3 represents the genetic profile characteristic of landraces and cultivars, emerging as the predominant configuration in contemporary agricultural systems.

The SiSRS4 clade contains pattern4_1 and pattern4_2, which exhibit differentiation patterns analogous to those between the previously described pattern5_2 and pattern5_3. Additionally, the SiSRS1 clade and SiSRS6 clade predominantly exhibit one distribution pattern, respectively. The SiSRS3 promoter displays the most complex variation.

### 3.4. Pan-Genome-Based Motif and Conserved Domain Analysis

The motif arrangement characteristics of the SiSRS family can be clustered into six major branches, designated as Type I–VI (Supplementary Figure S2A). Type I includes SiSRS1 members, with a conserved representative motif arrangement from the amino terminus to the carboxyl terminus as follows: motif 8-5-10-1-4-7-3-2-9-6. Notably, two sequence-length variations exist between motif 7 and motif 3, classified as Type I-L (50 accessions) and Type I-S (59 accessions). Type I-L exclusively contains two wild accessions (Q33 and Q36). Intriguingly, L14_SiSRS1 exhibits nearly duplicated sequence length and two nearly identical motif arrangements, with the first set lacking motif 6. This suggests a potential duplication event of *SRS* gene copies in the corresponding chromosomal region, where intervening sequences were lost during duplication. Type II is composed of SiSRS2 members, characterized by the motif order 7-8-9-5-1-4-3-2-10. Variations occur between motif 7 and motif 9 across different germplasms. Type III (SiSRS3) features the representative motif arrangement 9-5-1-4-7-3-2-10-8. However, 40 members display additional amino acid sequences preceding motif 9. Type IV (SiSRS4) exhibits two distinct motif arrangements differentiated by the presence or absence of N-terminal motif 9. Remarkably, among 73 accessions containing motif 9, only 7 are wild species (Q33, Q31, Q23, Q22, Q12, Q9, and Q2), while other wild SiSRS4 members lack this motif entirely. Type V (SiSRS5) displays the motif order 5-9-1-4-7-3-2-10-8. A unique duplication of motif 5 at the carboxyl terminus of motif 9 is observed in 39 landraces and cultivars and 12 wild accessions. The 9-10-5-1-4-7-3-2-8-6 motif arrangement constitutes the predominant organizational pattern in Type VI systems assembled by SiSRS6 members. All SiSRS members retain the DUF702 domain, a defining core feature of the SRS family (Supplementary Figure S2B). Strikingly, the Transposase_28 domain was identified in SiSRS5 members from three wild accessions (Q13, Q14, and Q18), suggesting potential transposon-mediated evolutionary events in these lineages.

### 3.5. SiSRS Family Members in xm and Yu1

**Figure 2:**
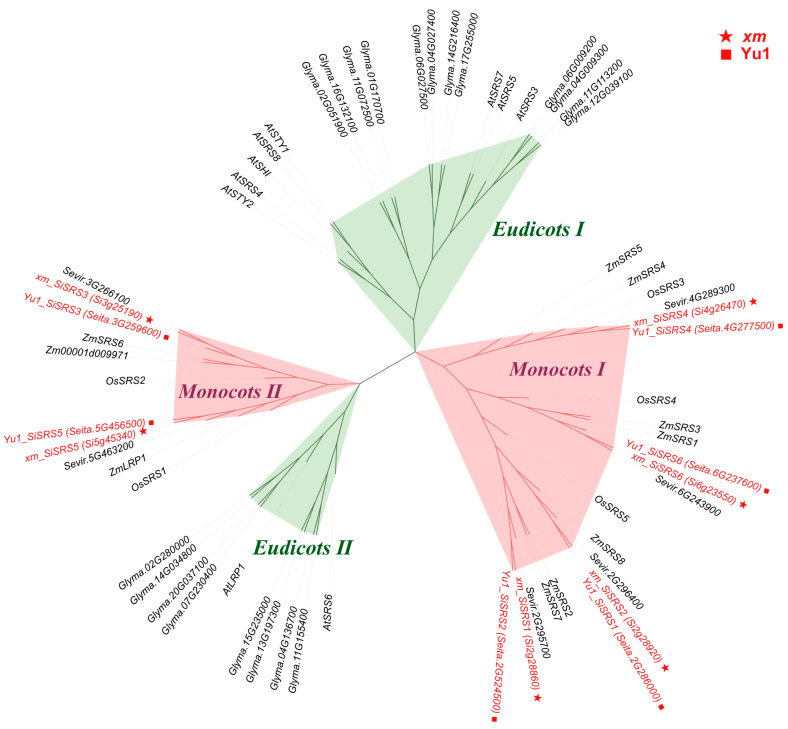
Phylogenetic analysis of the SRS family of *xm* and Yu1.

This study employed two representative foxtail millet accessions (*xm* and Yugu1), which possess high-quality genomes and comprehensive multi-omics datasets, to systematically investigate the characteristic patterns of *SiSRS* core family members. Both cultivars harbored six conserved *SiSRS* core members. Maximum likelihood phylogenetic reconstruction revealed that the entire SiSRS family can be divided into two major clades (Figure 2). Within each developmental clade, monocot and dicot *SRS* genes exhibit distinct independence, with the overall family being further subdivided into the following two groups: Monocots I and II and Eudicots I and II. Specifically, SiSRS3 and SiSRS5 are classified under Monocots I, including the LRP of *A. thaliana*, while SiSRS1, SiSRS2, SiSRS4, and SiSRS6 are grouped under STY/SHIs containing Monocots II. Additionally, *SiSRS1* and *SiSRS2* genes exhibit a cross-corresponding relationship between the *xm* and Yu1 varieties.

**Figure 3:**
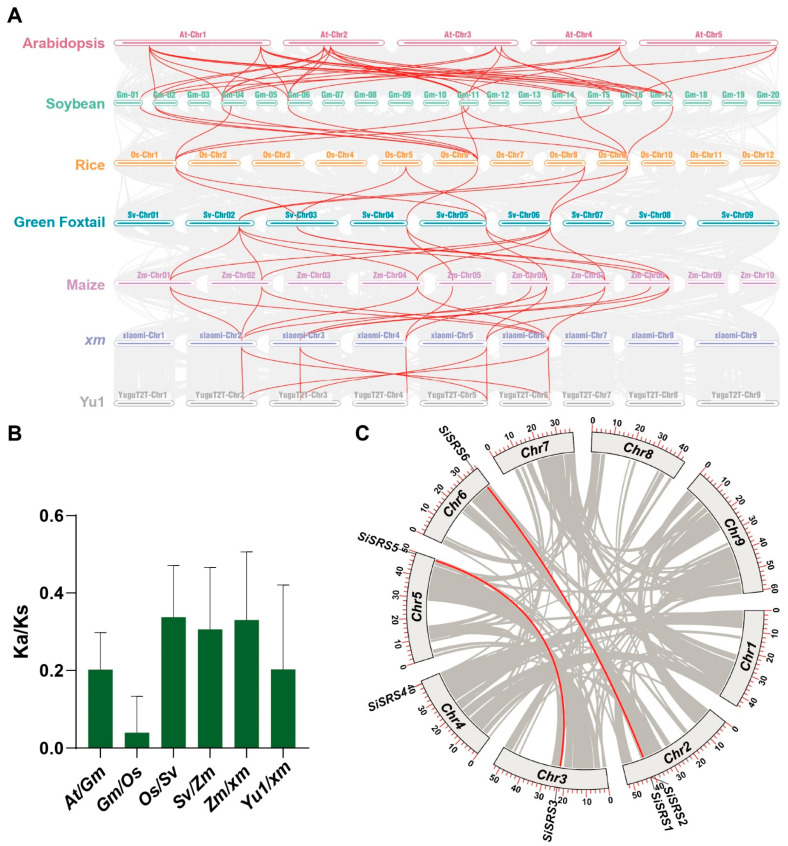
Evolutionary analysis of *SiSRS* members. (**A**) Synteny analysis of *SiSRS* members among reference species; (**B**) Ka/Ks analysis of recursive Synteny; (**C**) internal synteny analysis of the Yu1_SiSRSs.

Genome-wide collinearity analysis identified strong syntenic relationships between *SiSRS* genes and SRS homologs across five species (Figure 3A). For the number of collinear gene pairs in *A. thaliana*, soybean, rice, green foxtail, and maize were 7, 11, 5, 5, and 9, respectively (Figure 3A). Ka/Ks analysis of orthologous pairs revealed values < 1 across all comparisons (Figure 3B), consistent with purifying selection preserving core functional domains. Intraspecific collinearity in Yu1 further identified two paralogous pairs (*SiSRS1*-*SiSRS6* and *SiSRS3*-*SiSRS5*) with Ka/Ks < 1 (Figure 3C; Supplementary Table S3), reinforcing functional conservation under neutral evolutionary regimes.

The SiSRS protein family exhibits considerable variation in polypeptide length (226–411 amino acids), with molecular weights ranging from 22.98 to 42.06 kDa and isoelectric points (pI) spanning 7.70–9.10 (Supplementary Table S3). Protein sequence alignment confirmed that all members harbor signature features, including the C-X_2_-C-X_7_-C-X_4_-C-X_2_-C_2_-X_6_-C zinc-binding motif, IXGH motif, nuclear localization signal (NLS), and variable-length Q-rich region (Supplementary Figures S3 and S4).

### 3.6. SiSRS Promoter Architecture and Stress-Responsive Cis-Regulatory Element Profiling in xm and Yu1

**Figure 4:**
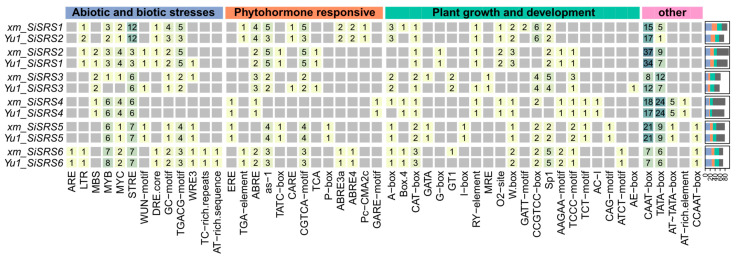
*Cis*-regulatory elements in *SiSRS* promoters of *xm* and Yu1. The light gray square indicates 0.

To elucidate the transcriptional regulation mechanisms, the promoter regions (2 kb upstream of initiation codon) of *xm*_*SiSRSs* and *Yu1_SiSRSs* were aligned (Supplementary Figure S5) and analyzed using PlantCARE (Figure 4). A total of 52 *cis*-elements were identified, categorized into four functional groups as follows: plant growth/development (21 elements), stress response (13 elements), hormone signaling (13 elements), and essential/core regulatory elements (5 elements). Among the most closely related gene pairs, except for *xm_SiSRS1* vs. *Yu1_SiSRS2* (81.40%) and *xm_SiSRS3* vs. *Yu1_SiSRS3* (85.61%), their promoter sequences exhibit extremely high homology (>99%), resulting in closely distributed regulatory elements. However, among *SiSRS* pairs, stress-responsive elements displays distinct distribution patterns. For instance, the environmental adaptation-related Myb element shows marked variability, with *xm/Yu1*_*SiSRS3* containing minimal counts (≤1) versus *xm/Yu1*_*SiSRS6* harboring the highest density (≥7 elements). Hypoxia/oxidative stress responsive GC-motifs are ubiquitously present, except in *xm/Yu1*_*SiSRS4*, peaking at *xm*_*SiSRS1* (4 elements) and *Yu1*_*SiSRS2* (3 elements). Stress response elements (STRE) playing a role in general stress response demonstrates broad distribution, with maximal enrichment in *xm*_*SiSRS1* and *Yu1*_*SiSRS2* (12 elements). Anaerobic response elements (AREs) localizes solely to *xm/Yu1*_*SiSRS6*, while drought-responsive MBS elements occurrs in *xm/Yu1*_*SiSRS2/3/4*. Wound-responsive WUN-motifs appears in *xm*_*SiSRS2/3/5*. LTR (Long Terminal Repeat) retrotransposon sequences were identified in *xm/Yu1*_*SiSRS1/2/6*. Hormone-specific elements P-box for gibberellin/drought response and light response elements (LREs) I-box are exclusively located in *xm/Yu1*_*SiSRS5*.

### 3.7. Expression Profiling of xm_SiSRS Genes in Salt and Drought Stress

**Figure 5:**
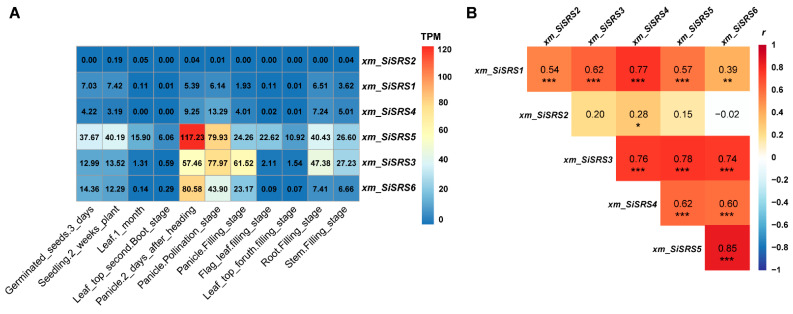
Transcription levels of *xm_SiSRSs* (**A**) and corresponding correlation analyses (**B**). *, *p* < 0.05; **, *p* < 0.01; ***, *p* < 0.001.

Transcriptomic analysis across developmental stages and tissues of *xm* revealed distinct expression patterns among *SiSRS* family members. *xm_SiSRS5* displayed the highest overall expression, with its peak transcript abundance (TPM = 117.23 ± 11.90) observed in panicles at 2 days post-heading (Figure 5A). Notably, *xm_SiSRS3* and *xm_SiSRS6* also exhibited pronounced expressions in panicles. During grain filling, *xm_SiSRS5* and *xm_SiSRS3* genes maintained elevated expressions in roots (40.43 ± 2.31; 47.38 ± 2.11) and stems (26.60 ± 13.80; 27.23 ± 10.70), while their leaf expressions remained comparatively low across all developmental phases. In contrast, *xm_SiSRS1, xm_SiSRS2,* and *xm_SiSRS4* showed minimal transcriptional activity with *xm_SiSRS2*, demonstrating near-undetectable expression levels (TPM ≤ 0.03 ± 0.05). Correlation analysis identified significant positive correlations between *xm_SiSRS1* and both *xm_SiSRS3* and *xm_SiSRS4* (*r* = 0.68–0.72, *p* < 0.001), while *xm_SiSRS3* exhibited strong associations with *xm_SiSRS4*/5/6 (*r* > 0.6, *p* < 0.001) (Figure 5B), suggesting coordinated regulatory mechanisms among these genes.

**Figure 6:**
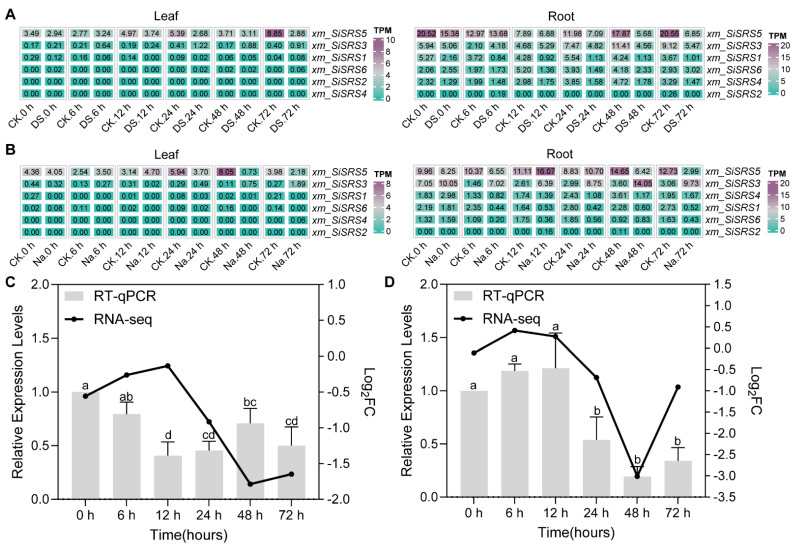
Transcription level analysis of *xm_SiSRS* genes under drought and salt stress. TPM variation in leaf and root tissues at different sampling times using RNA-seq analysis in (**A**) drought and (**B**) salt stress. The consistent analysis of *xm_SiSRS5* expression by RNA-Seq and RT-qPCR analysis in (**C**) drought-treated roots and (**D**) salt-treated leaves. The different letters above bars indicate statistically significant differences (*p* < 0.05). One-way ANOVA, n = 3.

Under abiotic stress conditions, RNA-seq profiling revealed tissue-specific responses. Salt (Na^+^) and drought (DS) treatments during seedling development induced significantly higher expression in roots than leaves (Figure 6A,B). *SiSRS5* emerged as the most stress-responsive member, with mean TPM values reaching 2.99 ± 1.30 at 72 h (Na^+^) and 5.68 ± 2.22 (DS) at 48 h in the root, compared to leaf values of 0.73 ± 0.31 at 48 h (Na^+^) and 2.68 ± 0.68 at 24 h (DS). Notwithstanding its secondary ranking in stress responsiveness, the *SiSRS3* gene exhibited low transcriptional activity, with TPM values hovering marginally above 1 throughout the experimental samples. Differential expression analysis (|Log_2_FC| ≥ 1, *p.adj* < 0.05) identified significant drought-induced downregulation of *SiSRS4* (24 h: Log_2_FC = −1.47, *p.adj* = 0.032; 48 h: Log_2_FC = −1.62, *p.adj* = 0.011) and *SiSRS5* (48 h: Log_2_FC = −1.79, *p.adj* = 6.50 × 10^−4^; 72 h: Log_2_FC = −1.65, *p.adj* = 0.002) in roots (Supplementary Table S6). Salt stress triggered the progressive suppression of *SiSRS5* in both leaves (48 h: Log_2_FC = −3.01, *p.adj* = 2.22 × 10^−7^) and roots (72 h: Log_2_FC = −1.87, *p.adj* = 0.0008) (Supplementary Table S6). The *SiSRS3* gene showed sustained upregulation in roots starting from 6 h of the stress treatment (6 h: Log_2_FC = 2.89, *p.adj* = 5.81 × 10^−5^; 12 h: 1.53, *p.adj* = 0.043; 24 h: 1.61, *p.adj* = 0.033; 48 h: 2.04, *p.adj* = 0.0046; 72 h: 1.87, *p.adj* = 0.0084). qRT-PCR validation also confirmed the trends of the *SiSRS5* gene, demonstrating significant root-specific downregulation after 12 h of drought (2.8-fold decrease, *p* < 0.01) and leaf-specific suppression (3.1-fold decrease, *p* < 0.05) following 24 h of salinity stress, consistent with transcriptome predictions (Figure 6C,D). However, the upregulation of the *SiSRS3* gene under salt treatment was not observed in the qRT-PCR results, which might be attributed to its lower transcription level.

### 3.8. Gene Co-Expression Network and PPI Analysis

**Figure 7:**
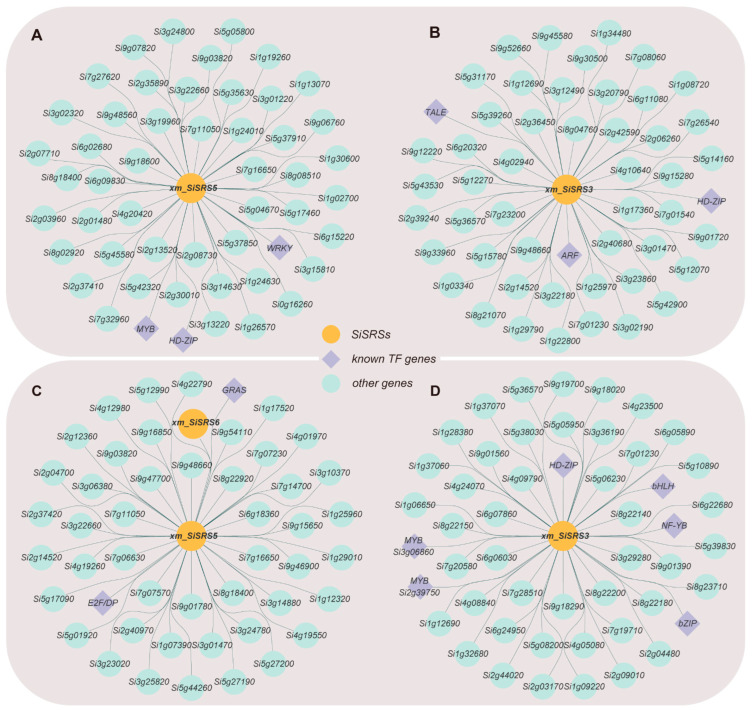
Gene co-expression network based on *xm* RNA-seq. The Orange circles represent SiSRSs; purple diamonds represent known TF-encoding genes, while cyan circles represent other genes. (**A**) Drought stress, *xm_SiSRS5*; (**B**) drought stress, *xm_SiSRS3*; (**C**) salt stress, *xm_SiSRS5*; and (**D**) salt stress, *xm_SiSRS3*.

Under drought stress conditions, co-expression network analysis revealed the distinct modular organization of *SiSRS* family members. Specifically, *xm_SiSRS3*, *xm_SiSRS4*, and *xm_SiSRS6* were clustered within the turquoise module, while *xm_SiSRS1* and *xm_SiSRS5* were localized to the yellow module, with *xm_SiSRS2* uniquely assigned to the magenta module (Supplementary Figure S6A). Within the yellow module, *xm_SiSRS5* exhibited co-expression with key transcription factors including *WRKY* (*Si3g20060*), the homeodomain-leucine zipper (*HD-ZIP*, *Si4g03500*), and *MYB* gene (*Si5g12210*) (Figure 7A). The turquoise module featured robust associations between *xm_SiSRS3* and transcription factors *HD-ZIP* (*Si9g15590*), three-amino acid loop extension (*TALE*, *Si3g21560*), and the basic leucine zipper (bZIP) family member *ARF* gene (*Si5g01440*) (Figure 7B). Furthermore, *xm_SiSRS5* demonstrated strong correlations with genes functionally linked to auxin biosynthesis (*YUCCA* gene *Si5g05800*), cytoskeletal dynamics (Tubulin Beta, *TUBB*, *Si5g04670*), membrane transport (*PIN*, *Si0g16260*; *TIP aquaporin*, *Si1g26570*), redox regulation (peroxidase, *POD*, *Si5g17460*), floral organ primordia forming (polygalacturonase-inhibiting protein, *PGIP*, *Si2g35890*), and stress signaling (*zinc finger protein SPOP gene*, *Si7g32960*; *IST*, *Si2g30010*). Notably, *xm_SiSRS3* displayed co-expression patterns with auxin biosynthesis genes (*TAA1*, *Si5g12270*; *YUCCA* family members, *Si5g31170*), microtubule components (Tubulin Alpha, *TUBA*, *Si2g36450*), and *POD* (*Si1g03340*, *Si9g33960*), suggesting its role in integrating growth regulation and oxidative stress responses under drought conditions. PGIP homologs (*Si2g35890*) displayed stress-responsive expression patterns overlapping with *xm_SiSRS5*.

Under salt stress, the *SiSRS* family members were partitioned into two distinct co-expression modules. *xm_SiSRS2* was exclusively assigned to the grey60 module, whereas the remaining five members (*xm_SiSRS1*/*3/4/5/6*) co-localized within the turquoise module (Supplementary Figure S6B). In the turquoise module, *xm_SiSRS5* formed co-expression hubs with cell cycle regulators (*E2F/DP*, *Si5g27800*) and developmental regulator genes (*GRAS*, *Si2g37190*) (Figure 7C), while *xm_SiSRS3* showed extensive connectivity with transcription factors spanning multiple families as follows: *MYB* (*Si2g39750*, *Si3g06860*), *HD-ZIP* (*Si9g15590*), *bZIP* (*Si5g35710*), basic helix-loop-helix (*bHLH*, *Si7g12250*), and nuclear transcription factor Y subunit beta (*NF-YB*, *Si5g44110*) (Figure 7D). Functional annotations revealed co-expressed partners of *xm_SiSRS3* included the stress-responsive 2OG-Fe(II) oxygenase gene (*Si9g01560*), while *xm_SiSRS5* was linked to transporter genes (*MATE*, *Si9g47700*; *PIN*, *Si1g29010*), cell wall modification enzyme genes (*endo-β-glucanase*, *Si7g16650*), and ROS signaling components (*RBOH*, *Si5g17090*). Intriguingly, multiple *xm_SiSRS* members exhibited co-expression relationships with auxin biosynthesis genes (*YUCCA*, *Si5g31170*) and auxin-responsive loci (*IAA*, *Si3g37160*, *Si5g12990*), highlighting the conserved regulatory interfaces between SiSRS proteins and hormonal pathways under salt stress (Supplementary Figure S6).

PPI network analysis delineated the functional orthology relationships between Yu1_SiSRS members and *A. thaliana* proteins. Yu1_SiSRS1 and Yu1_SiSRS2 corresponded to SRS1, Yu1_SiSRS3 to SRS7, Yu1_SiSRS4/Yu1_SiSRS6 to SHI, and Yu1_SiSRS5 to LRP1. Network topology revealed the significant interactions between NGA3 (AT1G01030, NGATHA) and four Yu1_SiSRS members (Yu1_SiSRS1, Yu1_SiSRS2, Yu1_SiSRS4, Yu1_SiSRS6), whereas YUC4 (AT5G11320, YUCCA4) interacted with all members except Yu1_SiSRS5. The ubiquitin-related factor F6N18.11 (AT1G32730) emerged as a universal interactor across the SiSRS family. Notably, Yu1_SiSRS5 exhibited exclusive interaction with translation initiation factor EIF4B3 (AT4G38710), while Yu1_SiSRS3 uniquely associated with proteolytic regulator Q9LU10_ARATH (AT5G39830, DEG8), suggesting specialized functional diversification within the SiSRS family (Supplementary Figure S7).

## 4. Discussion

### 4.1. Pan-Genome Analysis as a Paradigm Shift in Gene Family Annotation

The emergence of pan-genomic approaches has fundamentally transformed population genomic studies by overcoming the limitations of single-reference genome frameworks. While foundational studies in model plants like rice [41] and *A. thanalia* [42] established the essential genomic resources, these individual-centric references inherently fail to capture species-wide genetic diversity. Recent advances in multi-sample whole-genome sequencing have enabled the systematic characterization of structural variations (SVs) and copy number variations (CNVs) across cereal crops such as wheat [43], maize [44,45], and sorghum (*Sorghum bicolor*) [46], revealing their critical associations with agronomic traits. This pan-genomic perspective not only facilitates gene family identification at population scales but also enables integrative analyses of transcriptional and structural diversity, as demonstrated in barley bHLH transcription factor family studies [47].

To address the growing need for standardized nomenclature in pan-genome studies, we propose a novel annotation system as follows: *Accession_genefamilyN[.n]_xDy or xTy.* This framework integrates the following three key elements: (1) germplasm identifier, (2) gene family classification, and (3) chromosomal localization of core/variable genes. By explicitly encoding spatial distribution and CNV patterns, this system enhances cross-study comparability while maintaining backward compatibility with traditional annotation methods.

### 4.2. Evolutionary Conservation and Functional Constraints of SiSRS Genes

Analysis of 112 foxtail millet accessions revealed six core *SiSRS* members with near-universal conservation. Notably, eight accessions exhibited reduced copy numbers (five members), a phenomenon guaranteed by high-coverage PacBio (91.1×) and Illumina (48.1×) sequencing that excludes technical artifacts [22]. The exceptional stability of *SiSRS1*—showing no CNVs across all analyzed genomes—suggests stringent purifying selection, potentially linked to its constitutive low expression and limited stress responsiveness. With the exception of a small subset of members, the polypeptide sequence-based phylogenetic relationships of SiSRS were further corroborated by the clustering analysis of their corresponding promoter sequences. Interestingly, the distribution of CREs within promoter regions directly confirmed sequence insertion or deletion events occurring during domestication in wild progenitors. A notable example was the transition from the ancestral pattern5_1 to the derived pattern5_3 observed in the promoter region of *SiSRS5*. These findings comprehensively demonstrate that pan-genomic data harbor more abundant variation information critical for precision breeding.

### 4.3. xm_SiSRS5 Is the Important SiSRS Member

The *SRS* family exerts pleiotropic effects on auxin signaling and exhibit functional redundancy [2]. The co-expression network analysis in the study revealed the coordinated expression between *xm_SiSRS* members and both *IAA* and *YUCCA* family genes, suggesting the evolutionary conservation of auxin regulatory modules. *xm_*SiSRS5, the member exhibiting the highest expression level, showed functional parallels with LRP1, supported by its co-expression with MYB (*Si5g12210*) and WRKY (*Si3g20060*) transcription factors. This interaction mirrors the direct targeting of *PuLRP1* by PuMYB40 and PuWRKY75 during adventitious root formation in *Populus ussuriensis* [48], indicating potential cross-species conservation in root developmental regulation. The co-occurrence of *xm_SiSRS5* with *PIN* genes suggests its involvement in auxin polar transport [49], a mechanism further corroborated by the ARF transcription factor gene (*Si5g01440*, linked to *xm_SiSRS3*) that modulates floral development through auxin-mediated pathways [50]. The PGIP gene (*Si2g35890*) also displayed expression patterns overlapping with *xm_SiSRS5*. The OsPGIP encoded by *OsFOR1 *regulates floral organ primordia through LRR-mediated protein interactions in rice [51]. This functional convergence implies that xm_SiSRS5 may integrate floral development with environmental adaptation, though our sampling limitation to 21-day seedlings precludes conclusions about expression peaks during heading stages.

Notably, *xm_SiSRS5* is also the only member which is negatively regulated under drought and salt stress, validated by both RNA-seq and qRT-PCR analysis, simultaneously. The co-expressed *RBOH* (*Si5g17090*) and *POD* (*Si5g17460*), key enzymes in regulating ROS homeostasis, would support the observation further. RBOH generate superoxide radicals that are converted to H_2_O_2_ spontaneously or by SOD. The cell wall-bound Class III PODs also contribute to apoplastic H_2_O_2_ production. H_2_O_2_ plays an important role in regulating stomatal closure in response to drought [52] and modulating Na^+^/K^+^ homeostasis in salt stress condition [53,54]. This dual association would position xm_SiSRS5 as a potential modulator of ROS signaling cascades during abiotic stress. The effects of *xm_SiSRS5* overexpression or knockout on host resistance, as well as the functional performance of other homologs in other accessions, still warrant further validation.

## 5. Conclusions

This study systematically analyzed the presence, CREs, and motif distribution of the SRS family in 112 foxtail millet samples. A novel nomenclature for pan-genome gene families was proposed based on these findings, effectively illustrating the diversity of copy numbers and chromosomal location shifts among gene family members. Further analysis indicates that *xm_SiSRS5* exhibits the highest expression during the vegetative growth stage and has the strongest response to drought and salt stress. Future research should validate the specific molecular mechanisms of *SiSRS5* and investigate its potential applications in enhancing crop stress resistance.

## Data Availability

Data are contained within the article and Supplementary Materials.

## Supplementary Materials

The following supporting information can be downloaded at: https://www.mdpi.com/article/10.3390/plants14081257/s1, Figure S1. *Cis*-regulatory elements profiling in promoters of 678 *SiSRS* genes; Figure S2. Conserved motif (A) and domain (B) analysis of of 678 SiSRS proteins; Figure S3. Conservation analysis of the C-X_2_-C-X_7_-C-X_4_-C-X_2_-C_2_-X_6_-C motif of the SRS family in *xm* and Yu1; Figure S4. The multi-sequence alignment of SiSRS members of *xm* and Yu1. The Q-rich region, C-X_2_-C-X_7_-C-X_4_-C-X_2_-C_2_-X_6_-C and IXGH motifs were indicated by blue box; Figure S5. Alignment analysis of *SiSRS* promoters of *xm* and Yu1; Figure S6. Gene co-expression network of *xm_SiSRS* genes constructed in drought (A) and salt (B) stress; Figure S7. Protein-Protein Interaction network of xm_SiSRS proteins; Table S1. The distribution of SiSRS in the foxtail millet pan-genome; Table S2. BLASTP results of all SRS members in the pan-genome against the T2T genome; Table S3. Physicochemical property analysis of SRS proteins from *xm* and Yu1; Table S4. Ka/Ks analysis of *SiSRS* collinear gene pairs based on NG86 logic; Table S5. The component classification, names, and functions of *cis*-acting elements analysis; Table S6. Fold Changes of *SiSRS* genes under two stress conditions and two tissue types.
