# Supplementary material for: Pan-Genome-Based Characterization of the SRS Transcription Factor Family in Foxtail Millet
Source: Plants (Basel). 2025 Apr 21;14(8):1257. doi: 10.3390/plants14081257 (PMC12030303; doi:10.3390/plants14081257)
Supplement: Supplementary file 1 [file plants-14-01257-s001.zip › Supplementary Figure S3.pdf]

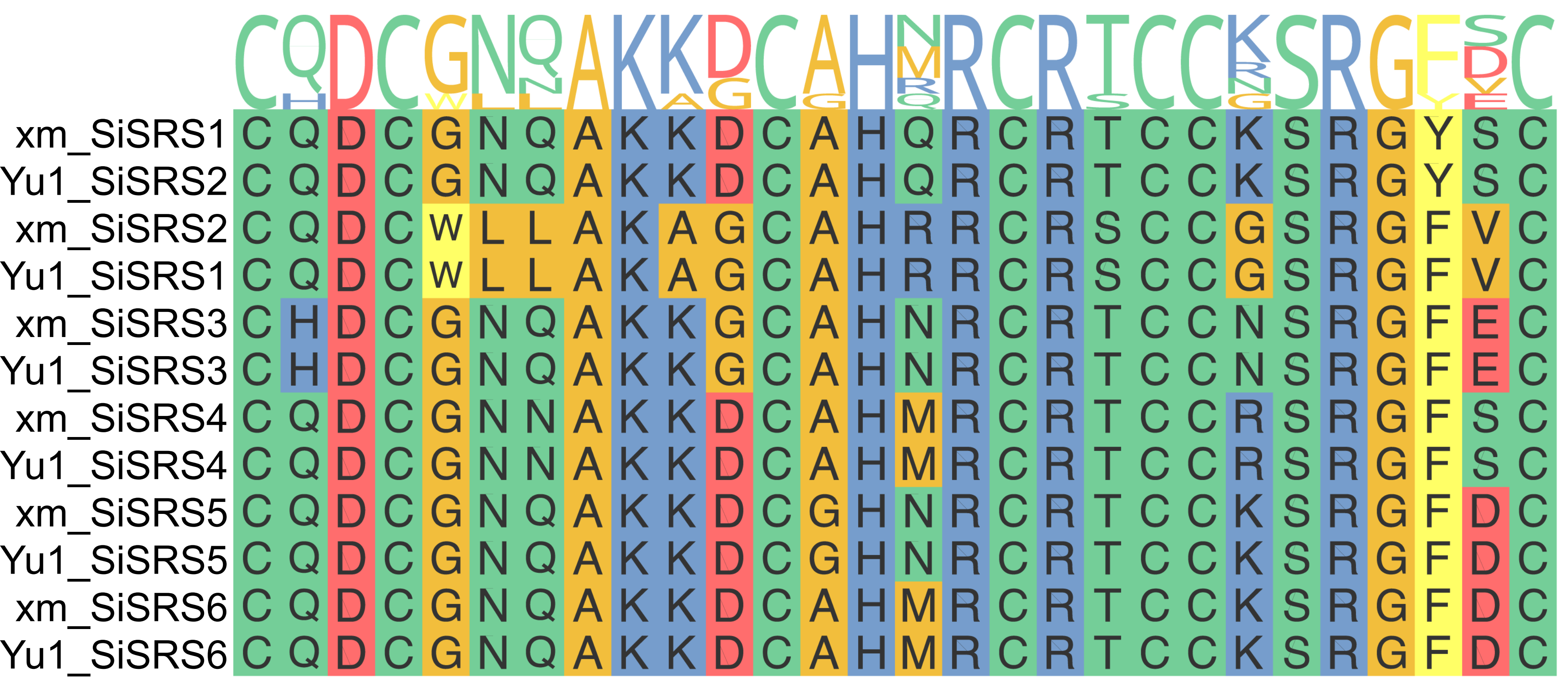
xm_SiSRS1 C Q D C G N Q A K K D C A H Q R C R T C C K S R G Y S C
Yu1_SiSRS2 C Q D C G N Q A K K D C A H Q R C R T C C K S R G Y S C
xm_SiSRS2 C Q D C W L L A K A G C A H R R C R S C C G S R G F V C
Yu1_SiSRS1 C Q D C W L L A K A G C A H R R C R S C C G S R G F V C
xm_SiSRS3 C H D C G N Q A K K G C A H N R C R T C C N S R G F E C
Yu1_SiSRS3 C H D C G N Q A K K G C A H N R C R T C C N S R G F E C
xm_SiSRS4 C Q D C G N N A K K D C A H M R C R T C C R S R G F S C
Yu1_SiSRS4 C Q D C G N N A K K D C A H M R C R T C C R S R G F S C
xm_SiSRS5 C Q D C G N Q A K K D C G H N R C R T C C K S R G F D C
Yu1_SiSRS5 C Q D C G N Q A K K D C G H N R C R T C C K S R G F D C
xm_SiSRS6 C Q D C G N Q A K K D C A H M R C R T C C K S R G F D C
Yu1_SiSRS6 C Q D C G N Q A K K D C A H M R C R T C C K S R G F D C
