# Supplementary material for: Pan-Genome-Based Characterization of the SRS Transcription Factor Family in Foxtail Millet
Source: Plants (Basel). 2025 Apr 21;14(8):1257. doi: 10.3390/plants14081257 (PMC12030303; doi:10.3390/plants14081257)
Supplement: Supplementary file 1 [file plants-14-01257-s001.zip › Supplementary Figure S4.pdf]

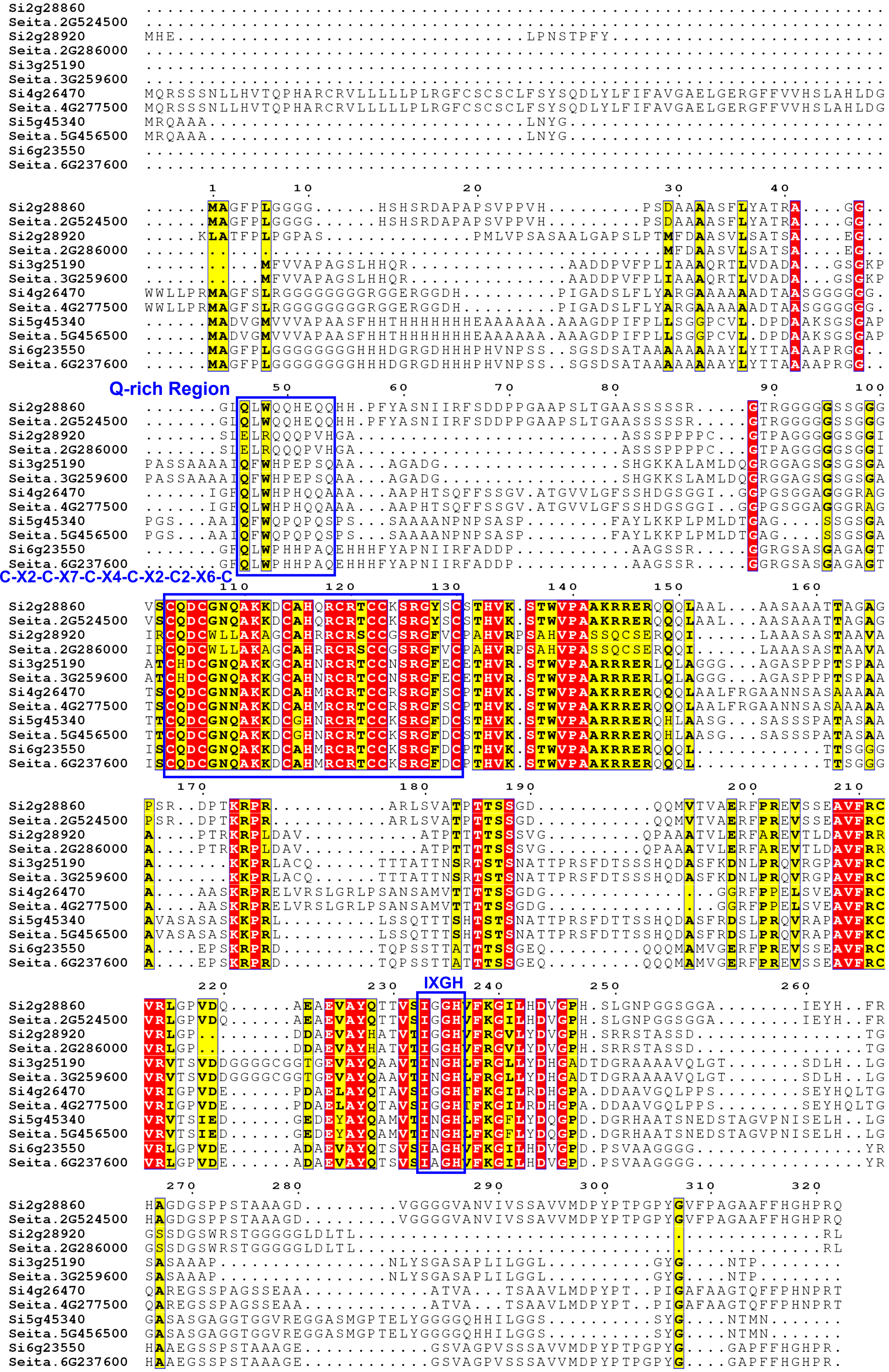
Q-rich Region
C-X2-C-X7-C-X4-C-X2-C2-X6-C
IXGH
Si2g28860
Seita.2G524500
Si2g28920
Seita.2G286000
Si3g25190
Seita.3G259600
Si4g26470
Seita.4G277500
Si5g45340
Seita.5G456500
Si6g23550
Seita.6G237600
