# Supplementary material for: Pan-Genome-Based Characterization of the SRS Transcription Factor Family in Foxtail Millet
Source: Plants (Basel). 2025 Apr 21;14(8):1257. doi: 10.3390/plants14081257 (PMC12030303; doi:10.3390/plants14081257)
Supplement: Supplementary file 1 [file plants-14-01257-s001.zip › Supplementary Figure_Legend.pdf]

Supplementary Figure S1 Cis-regulatory elements profiling in promoters of 678 *SiSRS* genes

Supplementary Figure S2 Conserved motif (A) and domain (B) analysis of of 678 SiSRS proteins

Supplementary Figure S3 Conservation analysis of the C-X_2-C-X_7-C-X_4-C-X_2-C2-X_6-C motif of the SRS family in *xm* and Yu1.

Supplementary Figure S4 The multi-sequence alignment of SiSRS members of *xm* and Yu1. The Q-rich region, C-X_2-C-X_7-C-X_4-C-X_2-C2-X_6-C and IXGH motifs were indicated by blue box.

Supplementary Figure S5 Alignment analysis of *SiSRS* promoters of *xm* and Yu1

Supplementary Figure S6 Gene co-expression network of *xm_SiSRS* genes constructed in drought (A) and salt (B) stress

Supplementary Figure S7 Protein-Protein Interaction network of xm_SiSRS proteins
