# Supplementary figures and images for: Pan-Genome-Based Characterization of the SRS Transcription Factor Family in Foxtail Millet
Source: Plants (Basel). 2025 Apr 21;14(8):1257. doi: 10.3390/plants14081257 (PMC12030303; doi:10.3390/plants14081257)

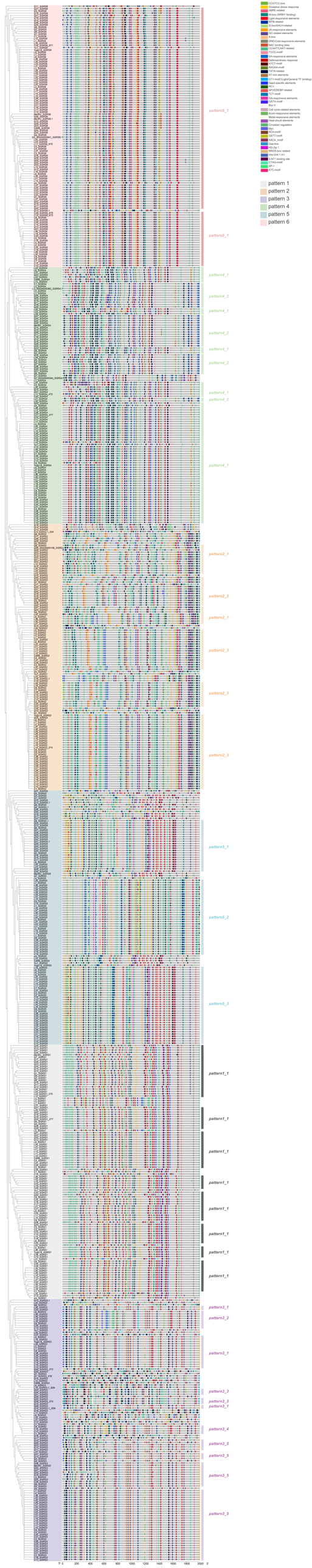

Supplement: Supplementary file 1 [file plants-14-01257-s001.zip › Supplementary Figure S1.pdf]

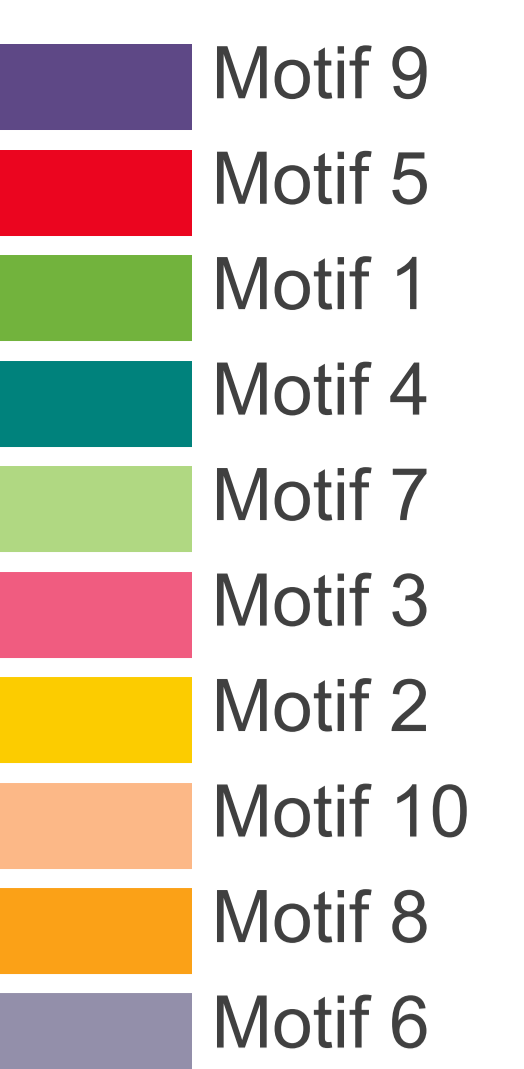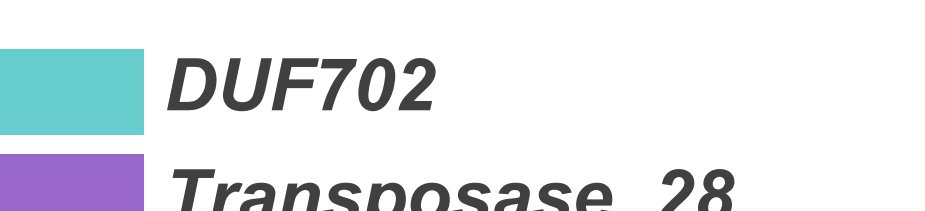

Supplement: Supplementary file 1 [file plants-14-01257-s001.zip › Supplementary Figure S2.pdf]

[illegible]

**yellow**

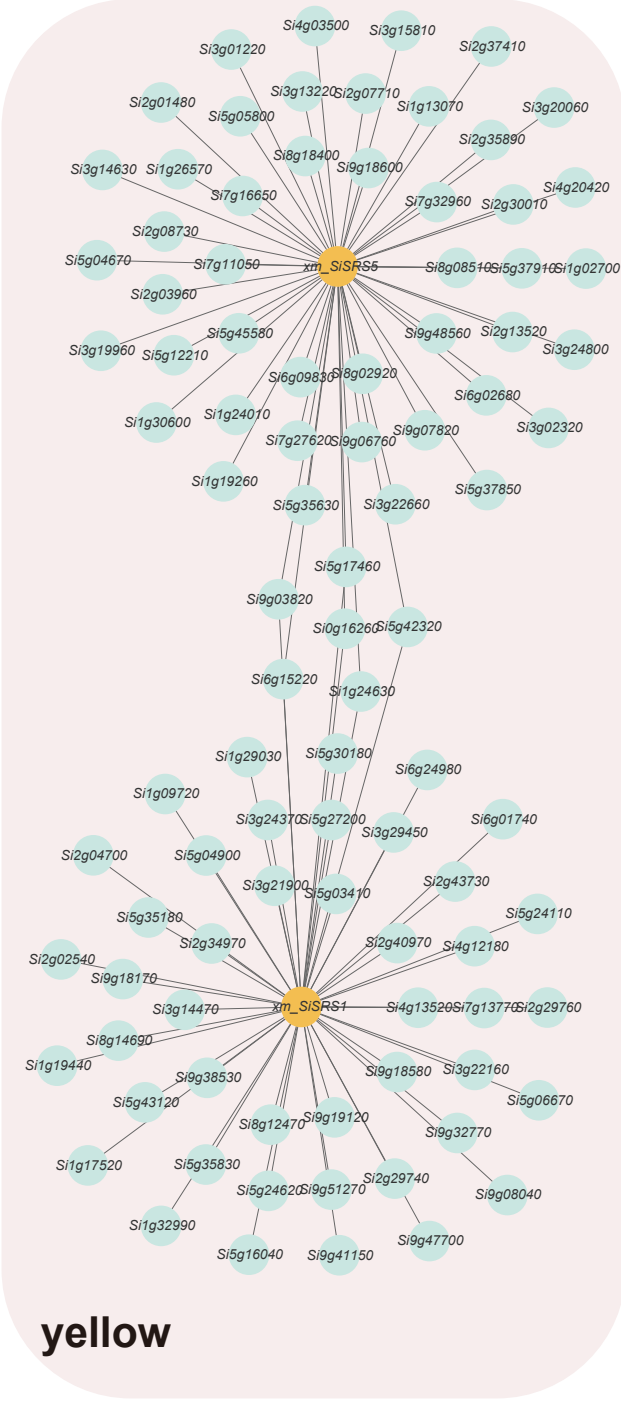

**B**

**grey60**

**turquoise**

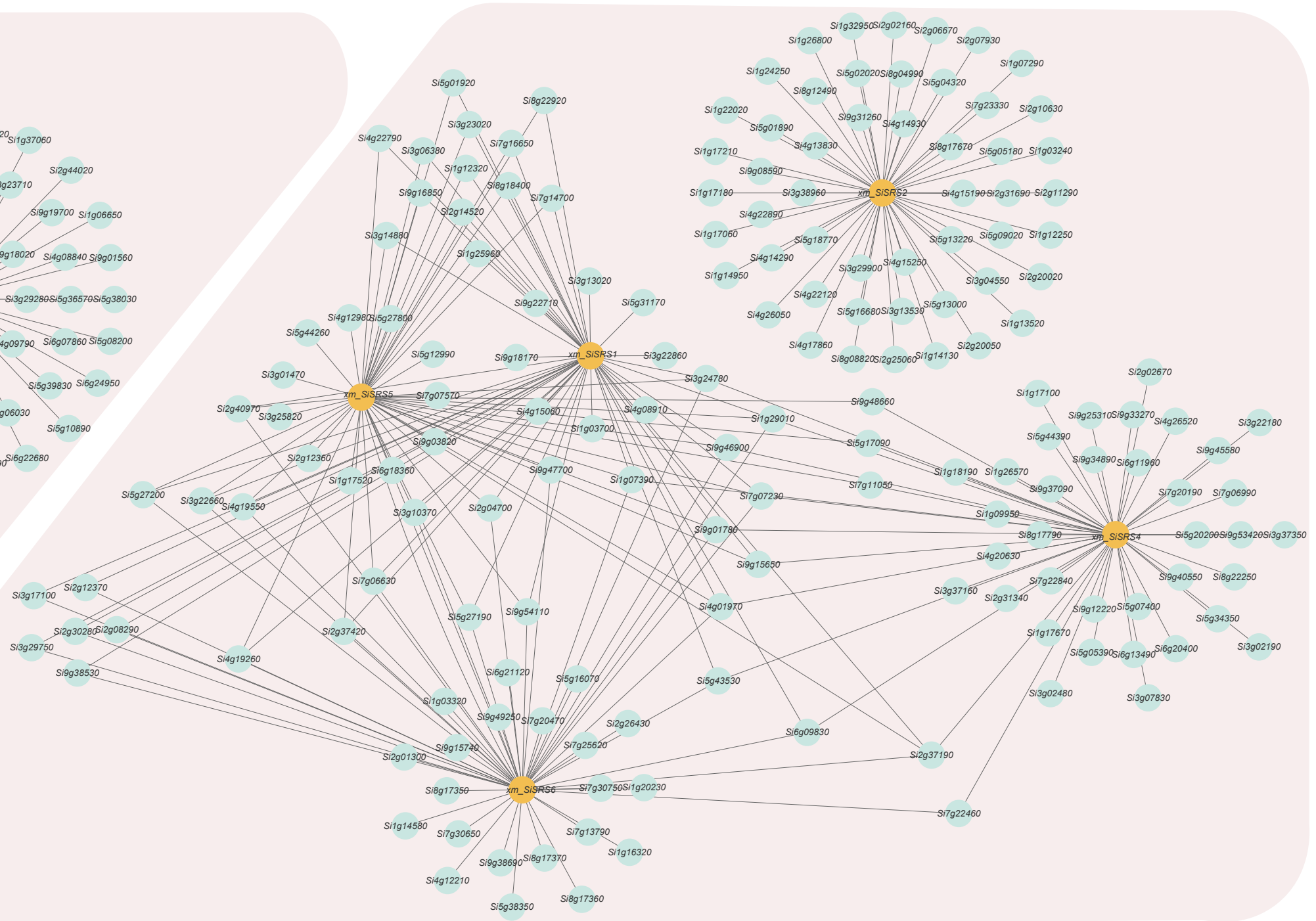

Supplement: Supplementary file 1 [file plants-14-01257-s001.zip › Supplementary Figure S6.pdf]

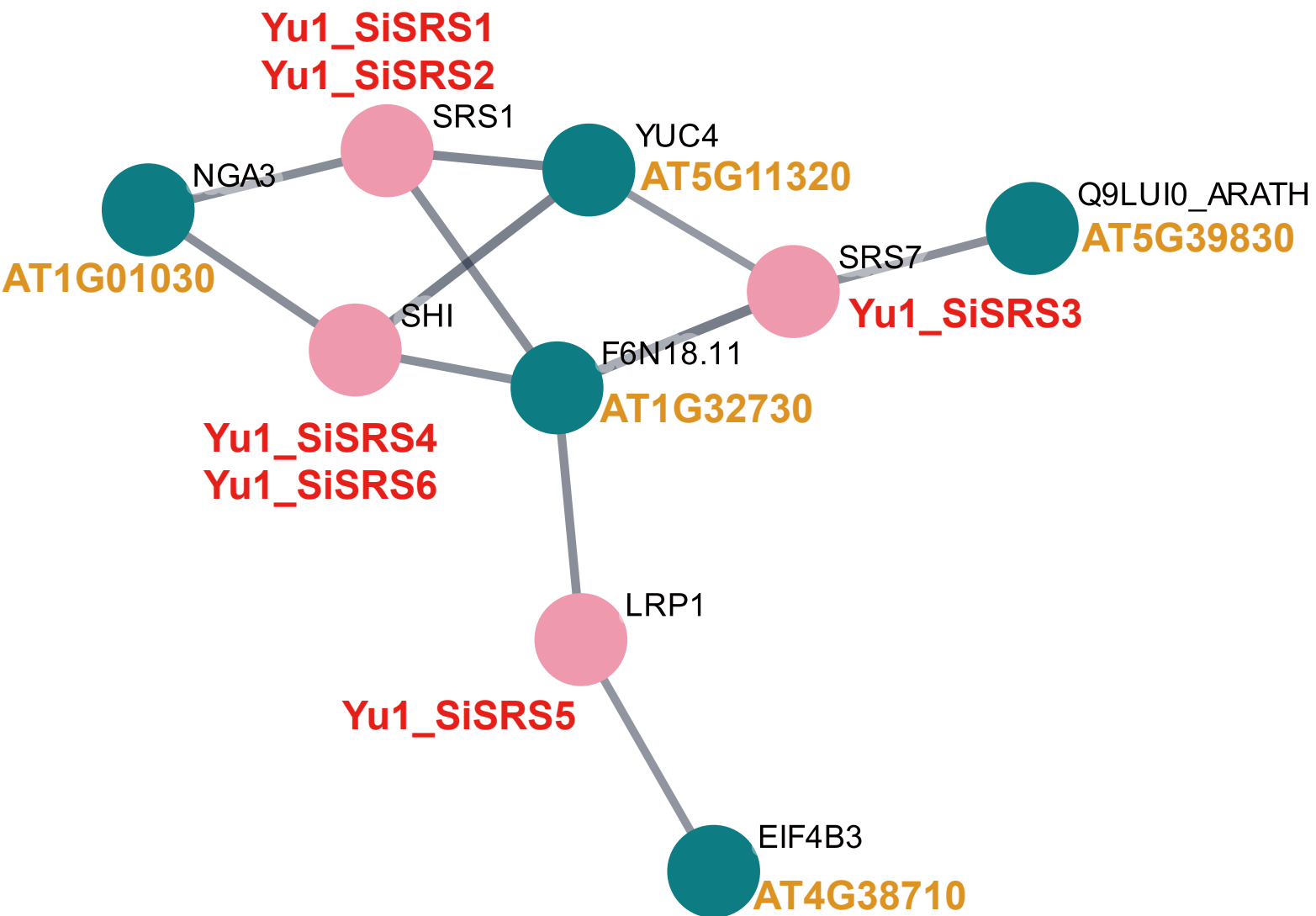

Supplement: Supplementary file 1 [file plants-14-01257-s001.zip › Supplementary Figure S7.pdf]
