# Supplementary material for: Pan-Genome-Based Characterization of the SRS Transcription Factor Family in Foxtail Millet
Source: Plants (Basel). 2025 Apr 21;14(8):1257. doi: 10.3390/plants14081257 (PMC12030303; doi:10.3390/plants14081257)

A

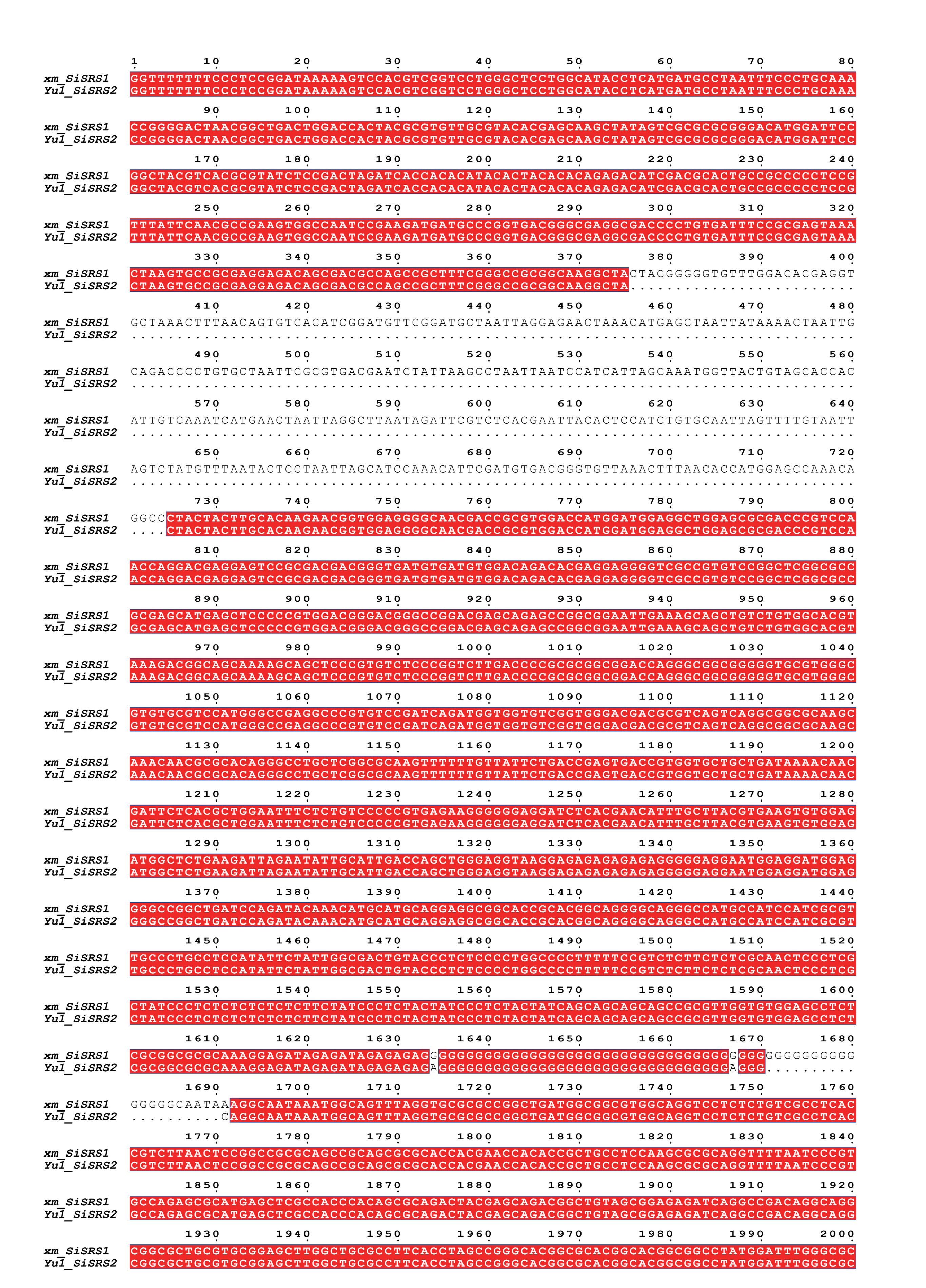

B

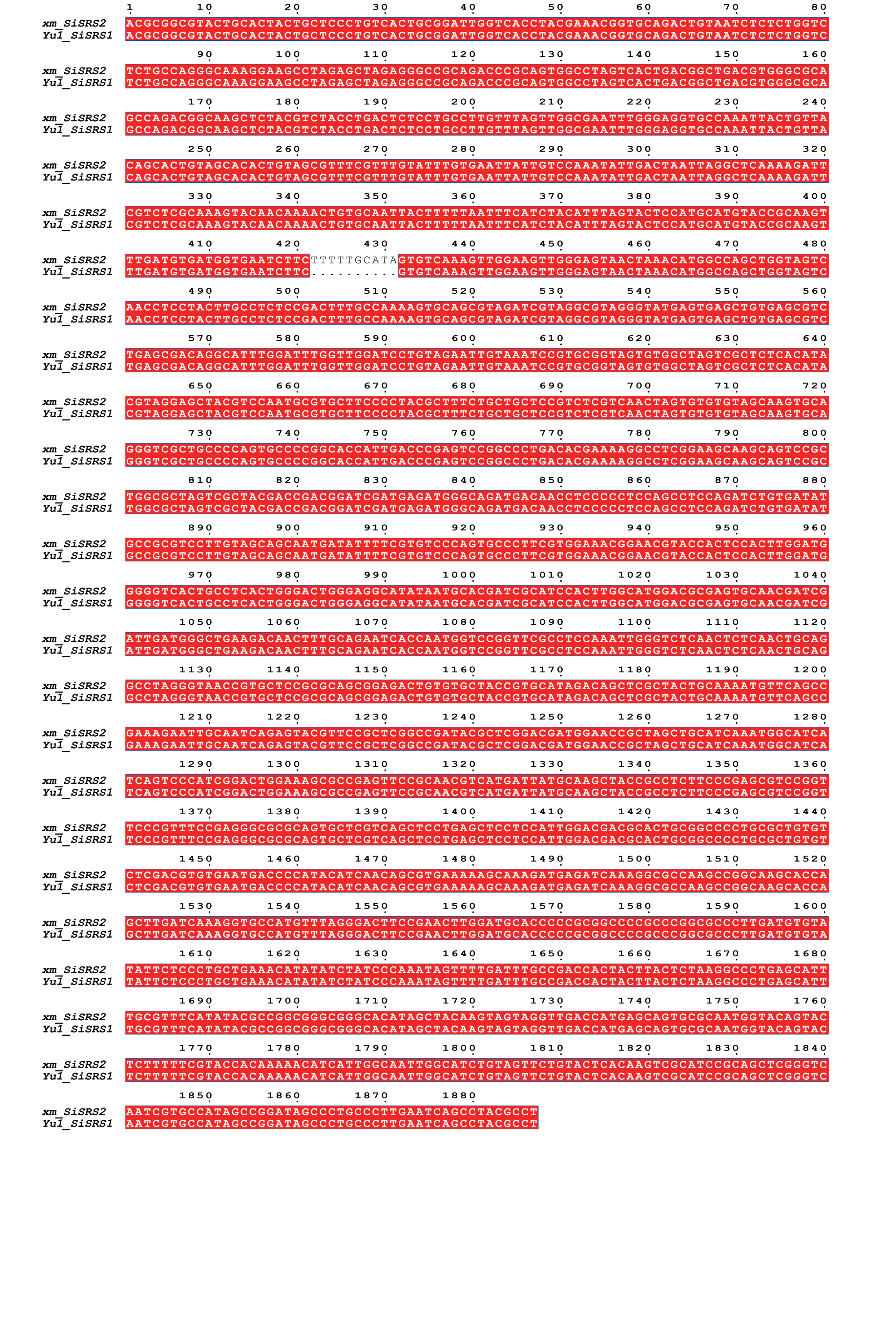

C

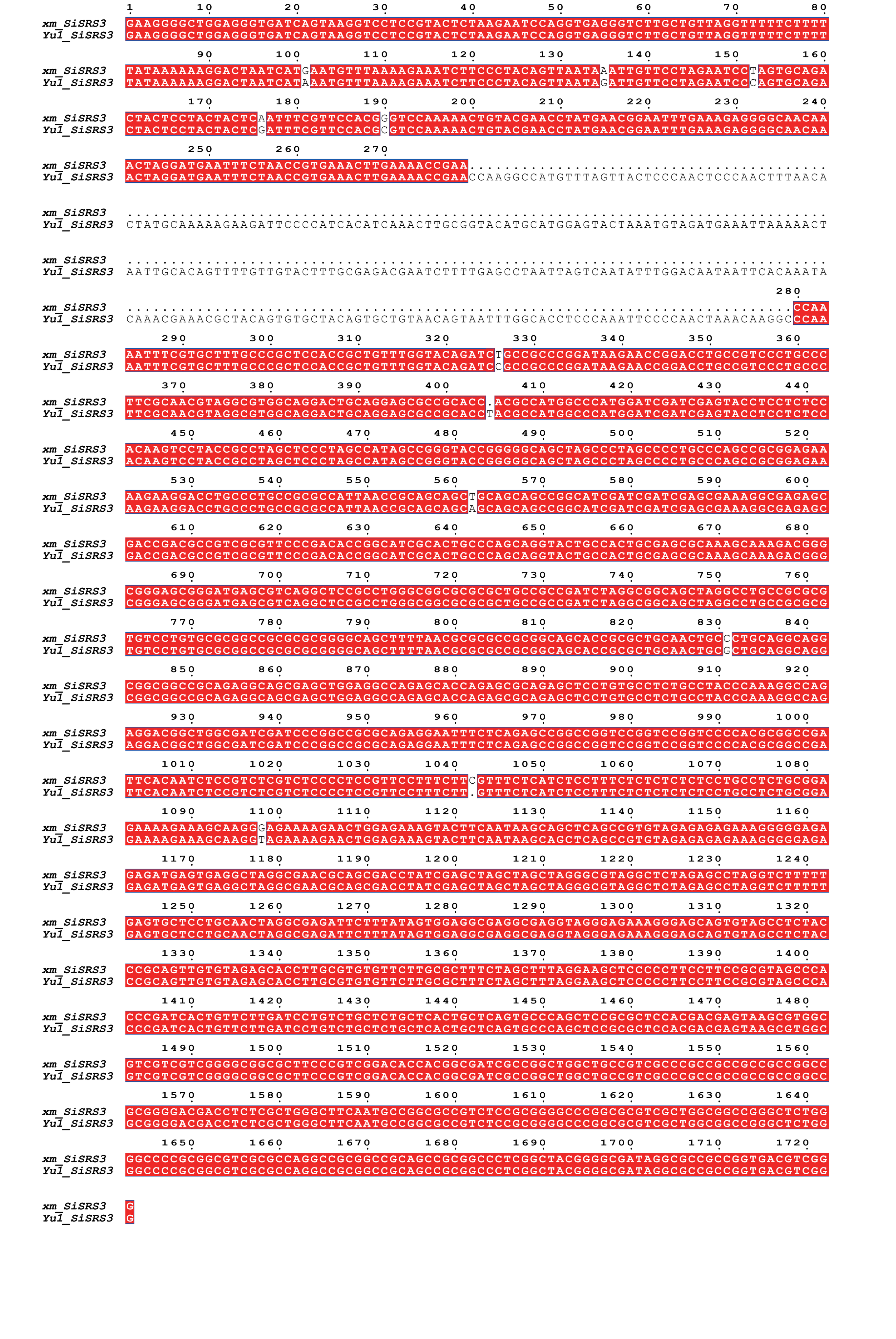

D

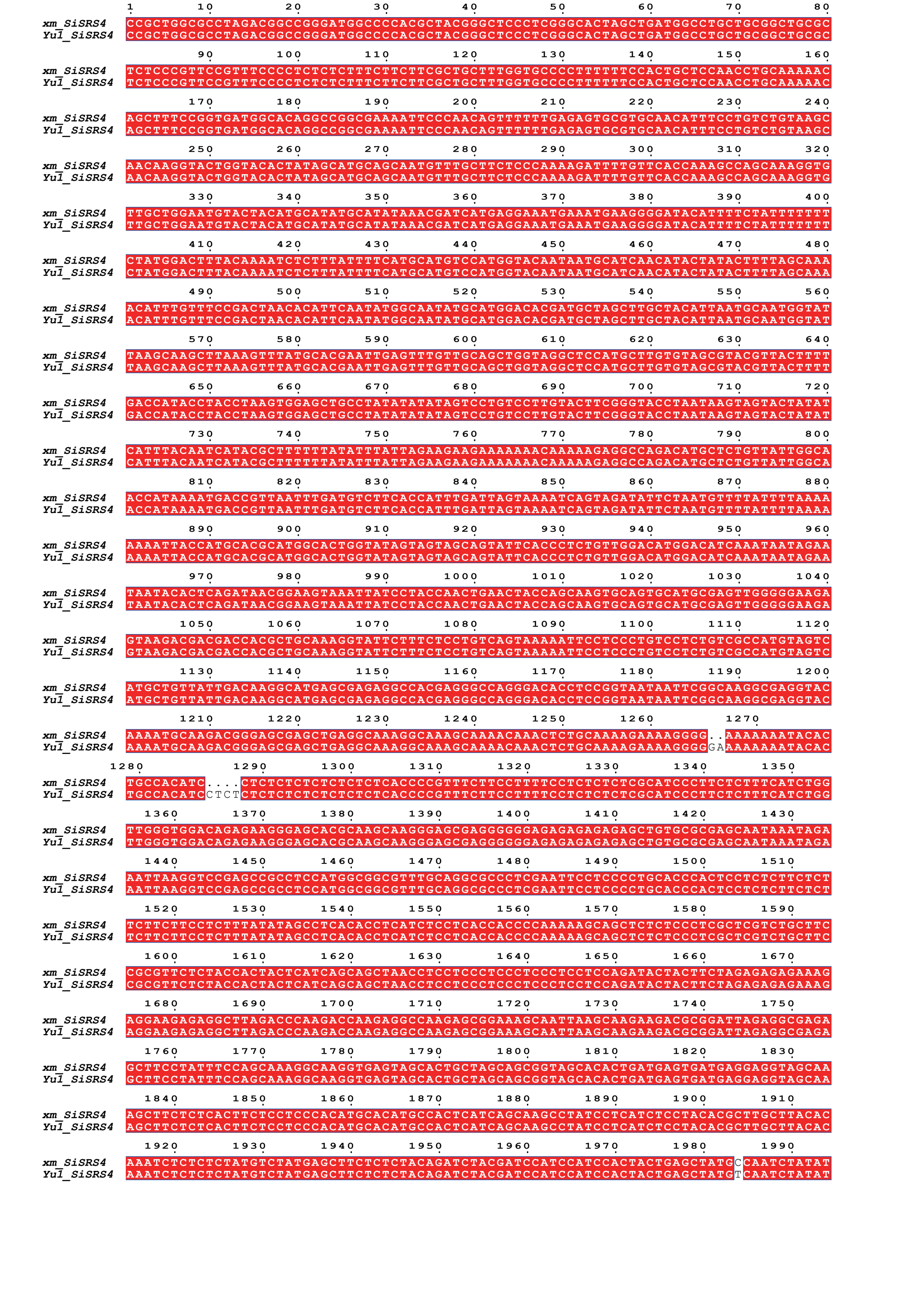

E

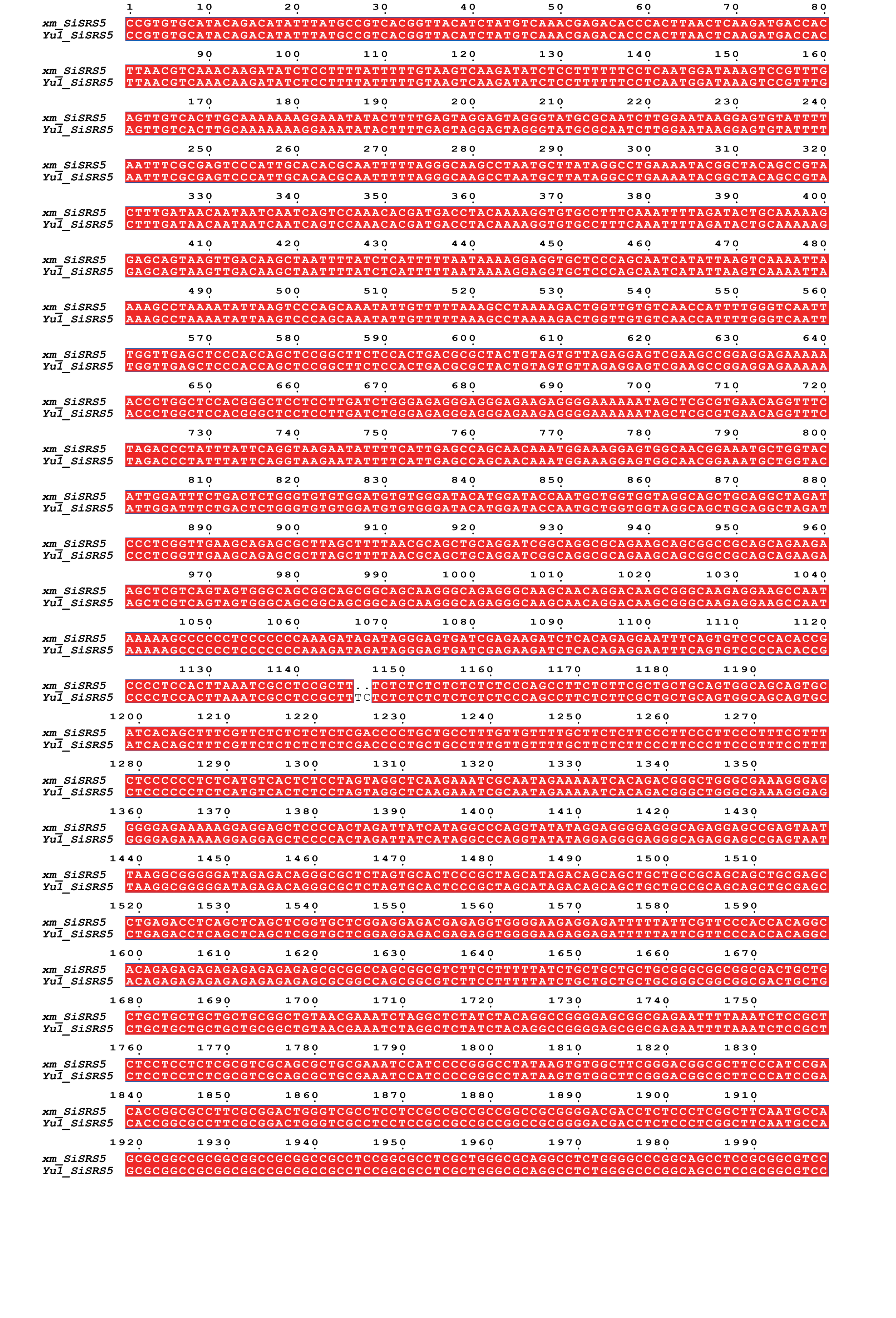

F

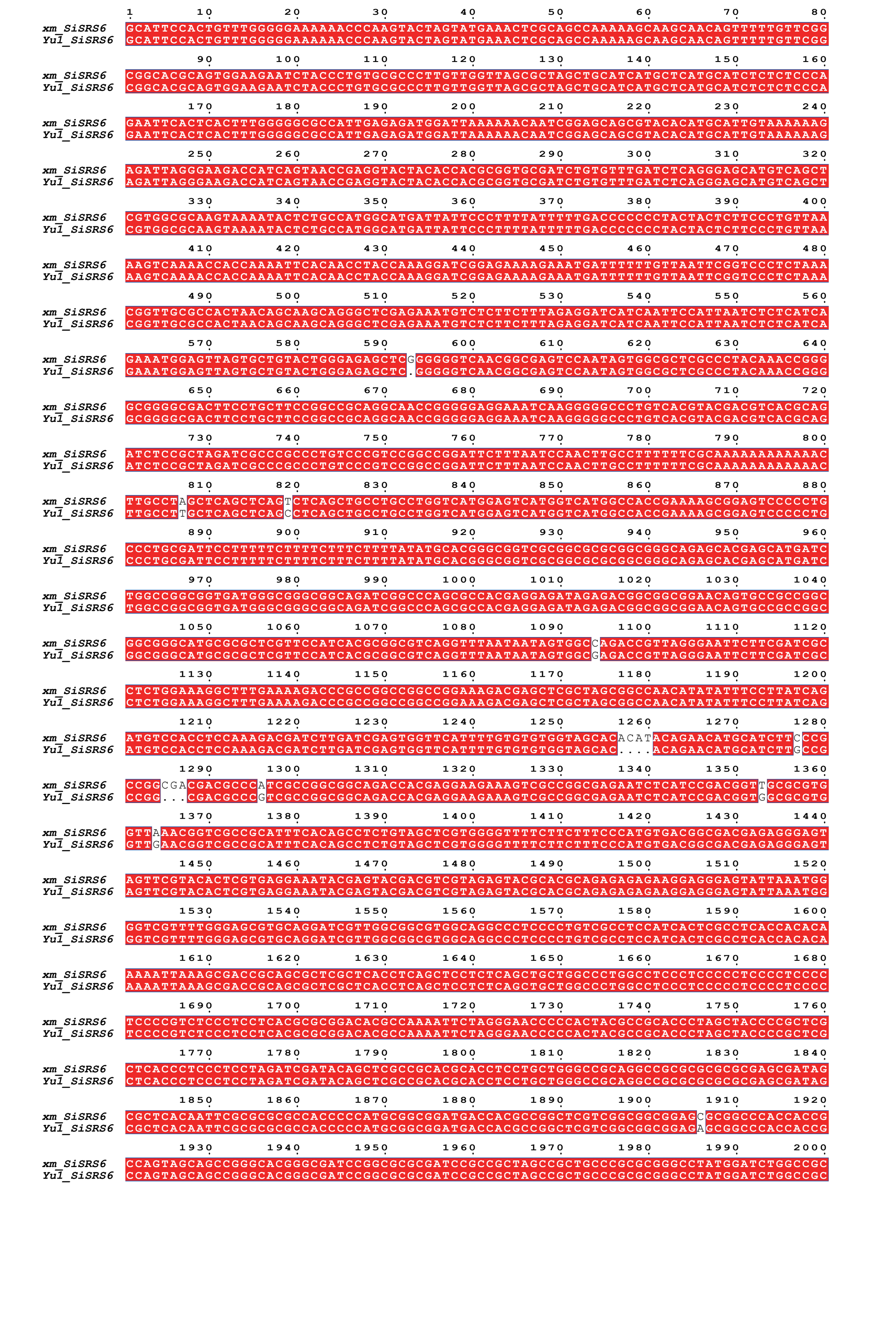

Supplement: Supplementary file 1 [file Supplementary Figure S5.pdf › Supplementary Figure S5.pdf]
